# Improving cell-free metabolism through direct integration of artificial respiratory chains

**DOI:** 10.1073/pnas.2613483123

**Published:** 2026-07-02

**Authors:** Owen D. Jarman, Nitin Bohra, Peter Claus, Nicole Paczia, Tobias J. Erb

**Affiliations:** ^a^ Department of Biochemistry and Synthetic Metabolism, Max Planck Institute for Terrestrial Microbiology, 35043 Marburg, Germany; ^b^ Max Planck School Matter to Life, 69120 Heidelberg, Germany; ^c^ Core Facility for Metabolomics and Small Molecule Mass Spectrometry, Max Planck Institute for Terrestrial Microbiology, 35043 Marburg, Germany; ^d^Center for Synthetic Microbiology, Philipps University Marburg, 35043 Marburg, Germany; ^e^Microbes-for-Climate Cluster of Excellence, Synmikro, 35043 Marburg, Germany

**Keywords:** synthetic CO_2_ fixation, proteoliposomes, artificial respiratory chains, energy modules, energy conservation

## Abstract

Energy-conserving mechanisms are essential in supporting cellular life. Yet in synthetic biology, it remains a challenge to reconstruct such processes from the bottom–up and integrate them with other biological functions to create complex systems with life-like properties. Recent efforts to build higher-order cell-free metabolic networks have suffered from the fact that their central oxidation reactions are not coupled to energy conservation, causing kinetic and thermodynamic limitations. Here, we developed an artificial respiratory chain that we tailored to sustain rapid electron transfer in a CO_2_-fixing 16-enzyme catalytic cycle (crotonyl-CoA/ethylmalonyl-CoA/hydroxybutyryl-CoA), while also exploiting the concurrent electron flow for adenosine triphosphate synthesis. We demonstrate how such artificial respiratory chains can be further diversified to accept multiple electron entries and coupled to other biological functionalities, such as cell-free transcription–translation networks. Altogether, our work highlights the opportunities and challenges of directly integrating energy conservation mechanisms when building toward self-sustaining/self-energizing artificial life-like systems.

## Significance

A key goal of bottom–up synthetic biology is to construct cell-free systems with life-like, autonomous, and self-sustaining capabilities. Achieving this requires an efficient and controllable energy supply. In this work, we integrate a custom-engineered proteoliposome-based energy module, enabling sustained adenosine triphosphateproduction to power complex metabolic processes. Crucially, our design conserves energy from otherwise wasteful oxidative metabolic processes and delivers a versatile platform for driving diverse cell-free networks. Our findings offer critical insights into the design of self-energizing metabolic systems, advancing efforts toward autonomous synthetic cells.

Life requires a finely tuned, recursive coupling of energy-consuming and energy-providing processes, which are linked through adenosine triphosphate (ATP), the primary energy currency of cells. In biological systems, energy is typically provided through oxidative metabolism, where electrons are channeled into a membrane-bound respiratory chain that converts the redox potential energy into an electrochemical gradient. This gradient is subsequently harnessed for ATP synthesis that in turn can be used for biosynthesis. Cells exploit multiple entry points for electrons to enter the respiratory chain, either directly, via membrane-bound substrate oxidations (e.g., succinate dehydrogenase), or through universal electron carriers such as NAD(P)H and electron transfer flavoproteins (ETFs) (1–3).

In synthetic biology, the recursive coupling of metabolism with energy generation remains a significant challenge toward developing self-energizing or autonomous systems with life-like functionalities. A variety of approaches have been taken over the years to artificially produce ATP, including direct enzymatically driven phosphorylations (4–6), electrobiological approaches (7) and through generating electrochemical gradients across membranes to drive ATP synthase activity (8–13). The latter approaches aim to mimic natural membrane-based ATP generation processes and are attractive for efforts to build complex metabolic pathways that can take advantage of unique membrane-based biochemistries, as well as membrane compartmentalization (14–16).

Some progress has been made recently by integrating natural membranes containing light-driven ATP synthesis machinery with cell-free systems—such as DNA transcription (17) or synthetic CO_2_ fixation cycles (13). However, these hybrid systems inherently limit engineering flexibility, because they still rely on preexisting membrane environments and host machineries. Alternative approaches involve the use of bottom–up proteoliposomes, that is, minimal membrane vesicles reconstituted with purified membrane proteins. For instance, light-driven proteoliposomes containing rhodopsin or photosystem II, coupled with ATP synthase, have been used to power cell-free transcription/translation (11), simple carbon fixation pathways (18) and actin polymerization (10). Similarly, “artificial mitochondria” have been reconstituted that are capable of driving simple metabolic processes from the supply of high-energy compounds (8, 12).

**Fig. 1.**
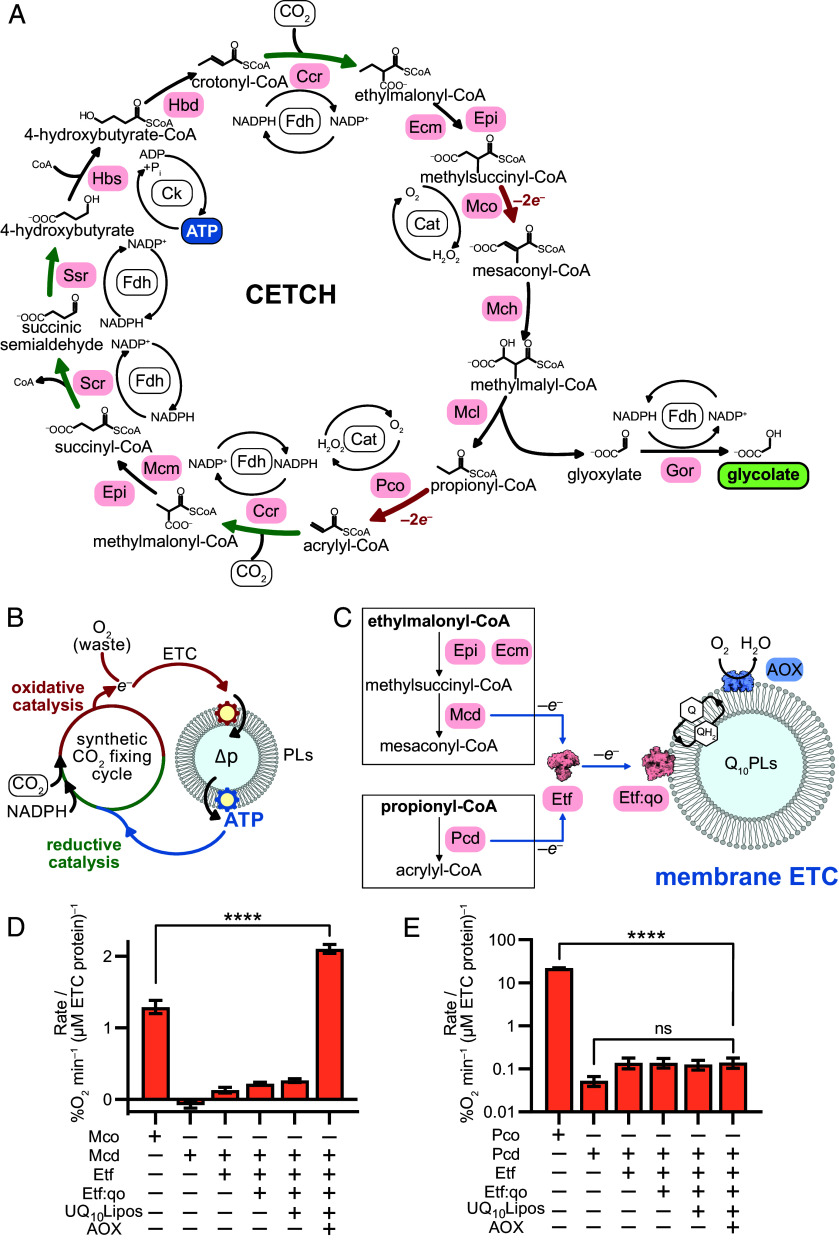
(*A*) Schematic overview of the full CETCH cycle reactions. Enzymes that catalyze each reaction, including ATP-dependent 4-hydroxybutyryl-CoA synthetase (Hbs) are shown. The two oxidation reactions are highlighted with red arrows and the reductive steps are highlighted with green arrows. The enzymes are abbreviated as shown in *SI Appendix*, Table S1. (*B*) Schematic of electron flow during artificial CO_2_ fixation. Electrons are transferred either directly to oxygen or through a membrane-bound respiratory chain, coupling the redox energy to ATP synthesis. Synthesized ATP can be used for anabolic reactions during CO_2_ fixation. (*C*) Schematic of assay conditions for oxygen consumption experiments. Electrons from the Mcd-catalyzed or Pcd-catalyzed oxidation step of the CETCH cycle are transferred through an artificial electron transport chain containing Etf, Etf:qo, UQ_10_-liposomes, and AOX. For the Mcd-catalyzed reaction, ethylmalonyl-CoA was used as a stable starting substrate so the labile methylsuccinyl-CoA was produced only during the assay measurement. For the Pcd-catalyzed reaction, propionyl-CoA was used directly. (*D*) Maximum rate of oxygen consumption during the oxidation of methylsuccinyl-CoA to mesaconyl-CoA per total concentration of protein (Mco vs. Mcd/Etf/Etf:qo:AOX) in each condition. Reactions were initiated with ethylmalonyl-CoA (100 µM), and methylmalonyl-/ethylmalonyl-CoA epimerase (Epi) and ethylmalonyl-CoA mutase (Ecm) were kept at a constant concentration throughout all conditions to convert ethylmalonyl-CoA into methylsuccinyl-CoA. The background oxygen consumption with only Ecm, Epi, and ethylmalonyl-CoA present is subtracted from all data. Shown are the average of at least three individually measured replicates ±SD. (*E*) Maximum rate of oxygen consumption during the oxidation of propionyl-CoA to acrylyl-CoA per total concentration of protein (Pco vs. Pcd/Etf/Etf:qo:AOX) in each condition. Reactions were initiated with propionyl-CoA (100 µM). The average background oxygen consumption with only propionyl-CoA present in the assay buffer is subtracted from all data. Shown are the average of at least three individually measured replicates ±SD. The concentration of all components in all panels are specified in *SI Appendix*, Table S2. Statistical significance in each panel was calculated by one-way ANOVA using Tukey’s test, ns *P* > 0.5, ****P < 0.0001.

Yet, in all these examples, the energy flow is still unidirectional, because an external energy source (e.g., light, reduced compounds) is provided “upstream” that powers “downstream” metabolic activities. Thus far, the recursive coupling of synthetic metabolic networks to artificial respiratory chains, creating interdependent, energetically efficient systems, remains an unrealized opportunity. One such case example is the crotonyl-CoA/ethylmalonyl-CoA/hydroxybutyryl-CoA (CETCH) cycle, a 16-enzyme in vitro CO_2_ fixation pathway that produces organic acids (e.g., glycolate) from CO_2_ (19) (Fig. 1*A*). This cycle features a reductive part that requires energy input in form of ATP and an oxidative part, in which energy is released through two acyl-CoA ester oxidation reactions. However, thus far, these oxidation reactions are kinetically limiting, and their energy is lost to H_2_O_2_ formation, which means that they cannot be harnessed to power the ATP-dependent reactions of the CETCH’s cycle reductive part. As a consequence, polyphosphate-, phosphocreatine-, as well as light-driven thylakoid-based ATP regeneration systems have been developed as external energy sources for the CETCH cycle (13, 19–21).

To overcome this fundamental limitation in powering synthetic metabolic systems, we sought to develop tailored artificial respiratory chains with the goal to achieve direct coupling between the in vitro metabolic network and energy modules (Fig. 1*B*). Exemplifying for the CETCH cycle, we show that such interlinked coupling of metabolism and energy modules enhance the cycle’s overall efficiency, both kinetically and thermodynamically. This interlinked coupling provides a step toward creating more complex systems with life-like properties, which we further exemplify by integration of a tailored artificial respiratory chain with cell-free information processing in a proof-of-principle.

## Results

### Establishing a Synthetic Electron Transport Chain.

To establish a link between the CETCH cycle and an energy supplying module, we sought to funnel electrons from the CETCH cycle’s acyl-CoA oxidation reactions through an electron transfer flavoprotein (Etf) to a membrane-bound Etf-quinone oxidoreductase (Etf:qo), establishing an ubiquinone (UQ) cycle with a proton-pumping ubiquinol oxidase that could be coupled to ATP synthase. Such link would salvage the otherwise wasted reduction potential of the electrons for the synthesis of ATP so it can be used to power the reductive part of the CETCH cycle (Fig. 1*B*).

In a first step, we sought to establish a basic conduit for electrons between the CETCH cycle and a membrane-bound platform electron transport chain (ETC) using proteoliposomes (PLs, Fig. 1*C*). For linking the CETCH cycle to the ETC, we initially focused on the oxidation of methylsuccinyl-CoA to mesaconyl-CoA. In the CETCH cycle, this reaction is catalyzed by an (engineered) methylsuccinyl-CoA oxidase (Mco), which donates extracted electrons directly to oxygen at a slow rate (Fig. 1*A*). Since Mco wastes chemical energy and requires high concentrations due to its low activity (Mco comprises 40 to 60% of all enzymes in CETCH cycle) (20), we sought to replace Mco with the native methylsuccinyl-CoA dehydrogenase (Mcd) from *Cereibacter sphaeroides* (22, 23). This enzyme transfers electrons via an Etf and a membrane-bound Etf:qo to the UQ pool, and from there to a terminal electron acceptor.

To establish a synthetic ETC in vitro, we prepared liposomes that resembled the phospholipid composition of *C. sphaeroides* membranes with a slight increased cardiolipin content [26:42:22:10 (% (w/w)) dioleoyl phosphocholine (DOPC): dioleoyl phosphoethanolamine (DOPE): dioleoyl phosphocholine (DOPG): cardiolipin (CDL)] (24). The liposomes were prepared with ubiquinone-10 (UQ_10_) for the UQ pool, and incorporated a short, artificial membrane-bound ETC, consisting of Etf:qo from *C. sphaeroides* and AOX (alternative oxidase) from *Trypanosoma brucei brucei* (25, 26), with oxygen as the final electron acceptor (Fig. 1*C*). Conveniently, both enzymes could be added in situ to the liposomes, naturally associating to the membrane without the need for detergent reconstitution. To couple the ETC to Mcd, we introduced *C. sphaeroides* Etf as an electron shuttle. When measuring the rate of oxygen consumption of methylsuccinyl-CoA oxidation (starting from ethylmalonyl-CoA, Fig. 1*C*), we observed low, but increasing oxygen consumption rates upon stepwise addition of each component of the ETC, up until the addition of AOX, where rates increased 1.63 ± 0.13-fold compared to Mco alone (Fig. 1*D*), indicating a functional ETC based on Mcd (Mcd:ETC).

We next explored replacing the second oxidation reaction in the CETCH cycle, i.e., the conversion of propionyl-CoA to acrylyl-CoA via propionyl-CoA oxidase (Pco) (Fig. 1*C*). To conserve energy of the propionyl-CoA oxidation step, we searched a suitable dehydrogenase that we could interface with our synthetic ETC, analogous to the Mcd reaction. We identified several candidates from *C. sphaeroides*, with a putative branched-chain acyl-CoA dehydrogenase (Pcd) showing some activity with propionyl-CoA (27). However, oxygen consumption measurements showed very low activity with no significant increase on implementing the complete ETC with AOX, suggesting that the final electron transfer reaction was no longer the rate limiting step, but likely the Pcd-catalyzed reaction itself (Fig. 1*E*). Enzyme engineering efforts did not further improve turnover or specificity of Pcd (*SI Appendix*, Fig. S1).

### Linking ETCs to the CETCH Cycle Improves Yields and Overcomes Kinetic Bottlenecks.

**Fig. 2.**
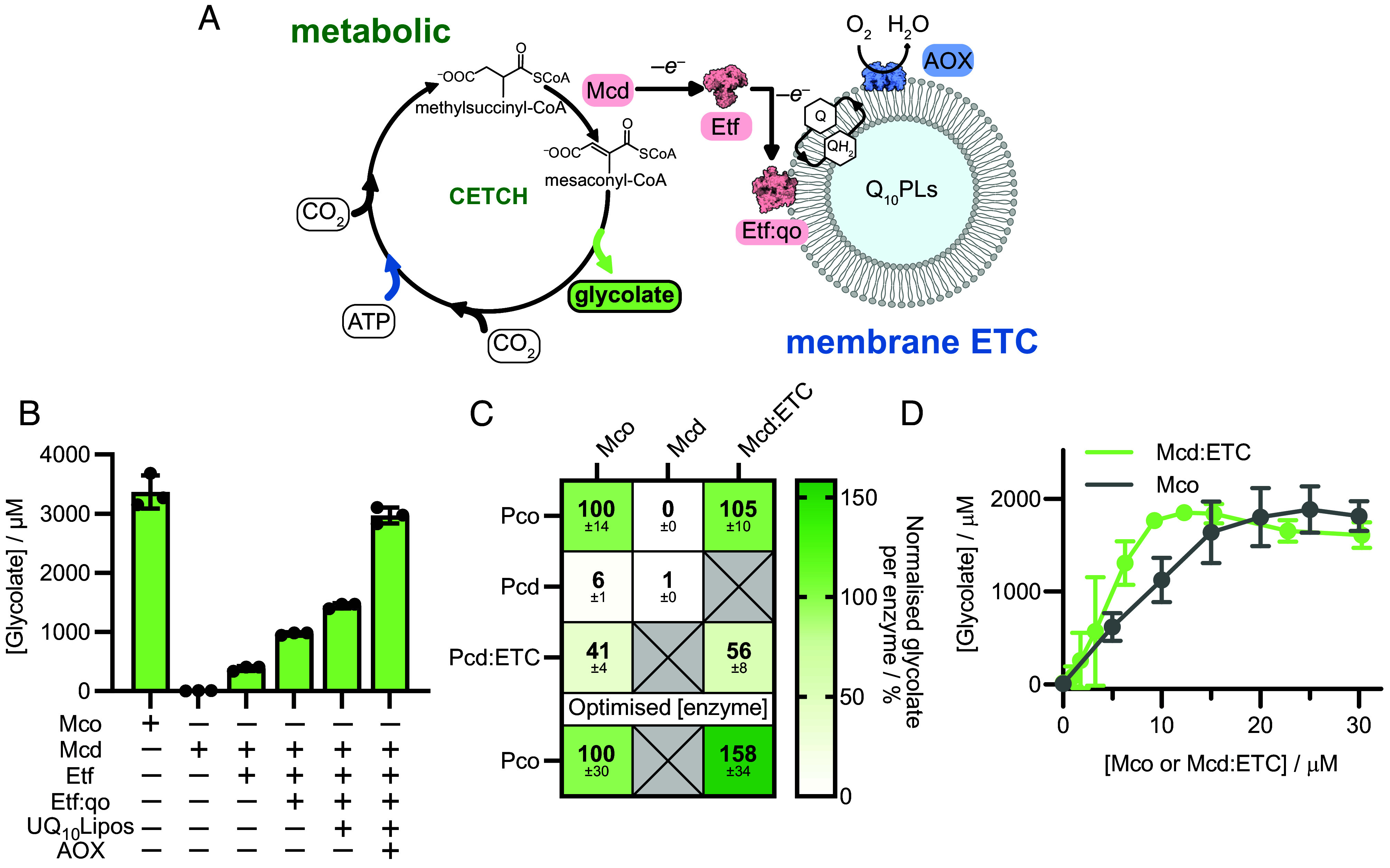
(*A*) Schematic of the methylsuccinyl-CoA oxidation step of the CETCH cycle. Electrons are donated through Etf to the membrane-bound Etf:qo and finally through a ubiquinone loop and the terminal oxidase AOX to oxygen. For a comprehensive representation of the CETCH cycle and enzyme abbreviations, see Fig. 1*A* and *SI Appendix*, Table S1. (*B*) Glycolate output of the Mco vs. Mcd:ETC-based CETCH cycles, after 4 h, upon addition of each ETC component and initiation with 100 µM propionyl-CoA. The concentration of all other components and enzymes were fixed. Data shown are the average of three technical replicates ±SD. For comparison of other CETCH cycle combinations, including Pcd:ETC vs. Pco-based cycles, *SI Appendix*, Fig. S2. (*C*) Comparison of the maximum glycolate output after 4 h for Mcd:ETC/Mco and Pcd:ETC/Pco-based CETCH cycle combinations. The glycolate output per total enzyme concentration of all enzymes in the CETCH cycle was calculated in each condition from data in panel *B* and *SI Appendix*, Fig. S2 and were normalized to the Mco/Pco-based version in each experiment. The values shown are the percentage glycolate yield per mol total CETCH cycle enzyme, relative to the Mco/Pco-based version. Data shown are the average of three technical replicates ±SD. For the optimized conditions of the Mcd:ETC/Pco-based CETCH cycle, the comparison was made at the following concentrations from panel *D*, where the greatest glycolate production differences were seen; Mco (10 µM) vs. Mcd (3 µM), Etf (3 µM), Etf:qo (3 µM), and AOX (0.26 µM). Statistical significance of the optimized condition was calculated by the unpaired *t* test, **P < 0.01. Combinations not measured are shown in gray. (*D*) Glycolate output of the CETCH cycle after 4 h with increasing concentration of Mco vs. the Mcd:ETC. The Mcd, Etf, and Etf:qo concentrations are increased concurrently from 0 to 15 µM and the AOX and UQ_10_-liposome concentration kept constant at 0.26 µM and 1 mg mL^–1^ respectively. Data shown are the average of three technical replicates ±SD. The concentration of all components in all panels are specified in *SI Appendix*, Table S2.

Having established functional links between the two acyl-CoA oxidation reactions of the CETCH cycle and our synthetic ETC, we next explored connecting a complete CETCH cycle with the ETC (Fig. 2*A*). To that end, we aimed at testing all four possible combinations of Mcd:ETC/Mco and Pcd:ETC/Pco-based CETCH cycle versions. We started with an Mcd:ETC/Pco-based CETCH cycle. Similar to the oxygen consumption assays, we saw an increasing output of the system (i.e., glycolate production from CO_2_), when reconstituting the ETC step-by-step (Fig. 2*B*). Critically, the fully reconstructed Mcd:ETC (4 µM Mcd, 8.26 µM ETC) produced glycolate at levels that were comparable to an optimized Mco/Pco-based CETCH cycle using Mco alone at 26 µM enzyme concentration (Fig. 2 *B* and *C*) (20). In contrast, when testing the Mco/Pcd:ETC-based CETCH cycle, the fully reconstructed Pcd:ETC (25 µM Pcd, 8.26 µM ETC) was not as productive as when using Pco alone at a much lower concentration of 3.1 µM, reaching 41 ± 4% of the glycolate yield per total enzyme concentration compared to the Mco/Pco-based cycle (Fig. 2*C* and *SI Appendix*, Fig. S2). This is in line with the hypothesis that the Pcd reaction is rate-limiting compared to the Pco reaction (Fig. 1*E*). However, when additionally coupling the Mcd (4 µM) reaction with the ETC (Mcd:ETC/Pcd:ETC), the yield per total enzyme compared to Mco/Pco increased to 56 ± 8% (*SI Appendix*, Fig. S2), suggesting the ability of the synthetic ETC to overcome kinetic bottlenecks of the Mco reaction.

In subsequent titration experiments of Mco and the entire Mcd:ETC, we determined that the Mcd:ETC/Pco-based version performed significantly better than a Mco/Pco-based version below a threshold concentration of ~15 µM Mcd:ETC, or Mco, respectively (Fig. 2*D*). Notably, at ~10 µM protein concentration, glycolate yield per mol total protein of the Mcd:ETC/Pco-based CETCH cycle increased significantly to 158 ± 34% compared to a corresponding Mco/Pco-based version (Fig. 1 *D* and *E*), even though we replaced a single oxidase (Mco) with an electron transferring cascade of four enzymes (Mcd, Etf, Etf:qo, and AOX). These results confirm the ability of our ETC to overcome apparent kinetic bottlenecks of the CETCH cycle. In summary, these results demonstrated that our synthetic ETC was functional with the CETCH cycle, and highlighted the advantages of employing synthetic ETCs in complex metabolic pathways to improve their overall performance.

### Expanding our ETC into an ATP-Regenerating Respiratory Chain.

**Fig. 3.**
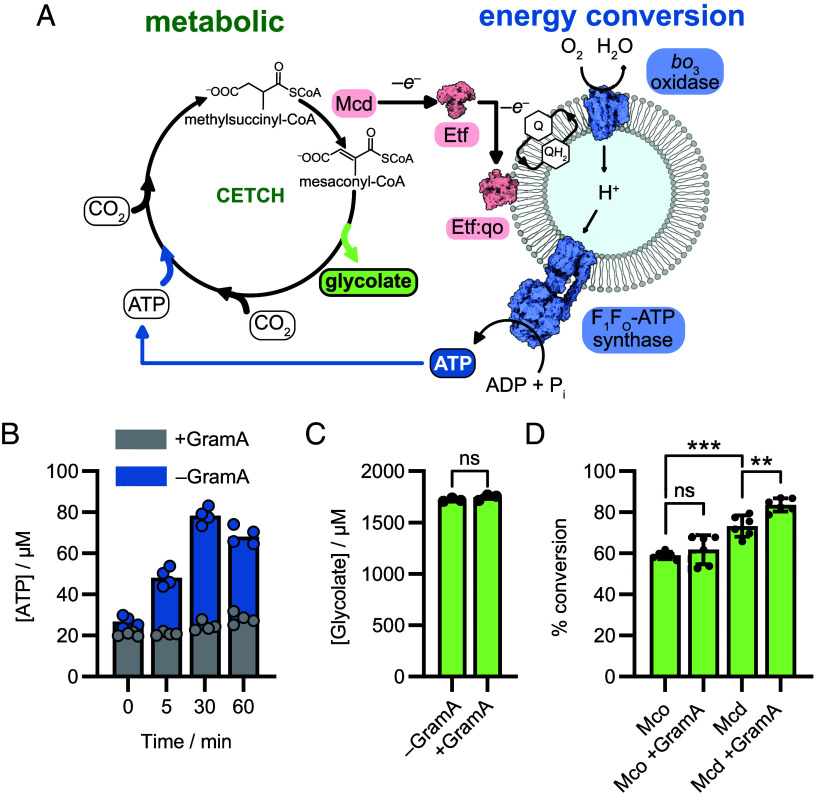
(*A*) Schematic of the Mcd-catalyzed acyl-CoA oxidation reaction of the CETCH cycle where electrons are shuttled through an artificial electron transport chain, first from Etf to Etf:qo. The quinone pool is reoxidized through the proton-pumping *bo*_3_ oxidase generating a proton motive force in the proteoliposomes. The F_1_F_O_-ATP synthase harnesses the proton motive force to synthesize ATP. (*B*) ATP synthesized from the Mcd-catalyzed oxidation step of the CETCH cycle conserved through an artificial respiratory chain (3 mg mL^–1^ Etf:qo-Cyt*bo*_3_-F_1_F_O_-PLs). Catalysis was initiated using 5 mM crotonyl-CoA and all enzymes were present to convert crotonyl-CoA through the oxidative side of the CETCH cycle to methylmalonyl-CoA. Methylsuccinyl-CoA (the substrate for Mcd) was not used directly as it is labile in solution. For a detailed breakdown of the reaction steps included in the assay, *SI Appendix*, Fig. S7. The protonophore gramicidin A (100 µg mL^–1^) uncoupled the membranes showing any nonspecific background ATP present in the assay setup. Data shown are the average of three technical replicates from four independent PL preparations ±SD. (*C*) Comparison of glycolate produced after 60 min in the same reaction conditions as panel *B*. Data shown are the average of three technical replicates from three independent PL preparations ±SD. Statistical significance was calculated by the unpaired *t* test, ns *P* > 0.5. (*D*) Conversion percentage of the complete CETCH cycle connected to the artificial respiratory chain (1 mg mL^–1^ Etf:qo-Cyt*bo*_3_-F_1_F_O_-PLs) without any additional ATP regeneration machinery (creatine kinase and creatine phosphate). Turnover was initiated on addition of 1 mM propionyl-CoA and ATP and glycolate production measured after 4 h incubation. Membranes were uncoupled with gramicidin A (100 µg mL^–1^). Percentage conversion was calculated from the measured glycolate concentration and the starting ATP/propionyl-CoA concentration. Statistical significance within each condition was calculated by one-way ANOVA using Tukey’s test, ns *P* > 0.5, **P < 0.01, ***P < 0.001. Data shown are the average of six technical replicates from two independent PL preparations ±SD. The concentration of all components are specified in *SI Appendix*, Table S2.

Next we aimed to extend our synthetic ETCs into ATP-generating respiratory chains. To achieve this, we reconstituted cytochrome *bo*_3_ ubiquinol oxidase (Cyt*bo*_3_) and F_1_F_O_-ATP synthase from *Escherichia coli* into liposomes containing UQ_10_ (Fig. 3*A*). For construction of liposomes, we adopted a phospholipid composition previously established to create artificial mitochondria (12), but supplemented with 1,2-dioleoyl-sn-glycero-3-phosphoglycerol (DOPG), a negatively charged lipid present in both *E. coli* and *C. sphaeroides* membranes. This phospholipid composition [60:20:10:10 (% (w/w)) DOPC:DOPG:DOPE:CDL] was used for all subsequent experiments. The resulting Cyt*bo*_3_-F_1_F_O_-PLs were able to generate a proton motive force across the membrane either via reverse ATP hydrolysis by F_1_F_O_-ATP synthase or *bo*_3_ oxidase catalysis initiated by short-chain ubiquinone (UQ_1_) reduced by dithiothreitol (DTT) (*SI Appendix*, Fig. S3).

Notably, in situ addition of Etf and Etf:qo to preestablished Cyt*bo*_3_-F_1_F_O_-PLs did not result in ATP production from ethylmalonyl-CoA oxidation (via Epi, Ecm, Mcd, Etf, Etf:qo). We hypothesized that membrane uncoupling occurred upon introduction of the detergent-purified Etf:qo (26, 28). Indeed, titrating Etf:qo into PLs that synthesized ATP through an independent oxidation module—that is NADH oxidation at NADH dehydrogenase (NDH-2) from *E. coli* (characterized in more detail later)—compromised ATP synthesis significantly at Etf:qo concentrations ≥0.5 µM (*SI Appendix*, Fig. S4) (29). Thus, we directly reconstituted Etf:qo together with Cyt*bo*_3_ and F_1_F_O_-ATP synthase into UQ_10_-liposomes, which resulted in the retaining of robust ATP generation through the NADH oxidation module (*SI Appendix*, Fig. S4). Critically, the reconstituted Etf:qo-Cyt*bo*_3_-F_1_F_O_-PLs were still viable as an ETC for the Mcd-catalyzed reaction as shown by comparable oxygen consumption rates to the nonreconstituted Etf:qo and AOX system (*SI Appendix*, Fig. S5).

Now, when coupling the Mcd oxidation reaction of the (partial) CETCH cycle to Etf:qo-Cyt*bo*_3_-F_1_F_O_-PLs, 54 ± 5 µM ATP was produced from 5 mM crotonyl-CoA within 30 min, about ~1% of the theoretical maximum ATP yield anticipated from the Mcd-catalyzed step (calculation in supplementary information) (Fig. 3*B* and *SI Appendix*, Fig. S6). To confirm that ATP production was dependent on the proton motive force, gramicidin A (GramA) was added to the liposomes, an ionophoric antibiotic that forms pores in the phospholipid bilayer, causing monovalent cations to diffuse across and depolarize the membrane. As expected, addition of the GramA lowered ATP production, while not affecting glycolate production (Fig. 3 *B* and *C*). In contrast, attempts to drive ATP synthesis through Pcd-catalyzed oxidation of propionyl-CoA (5 mM) did not result in GramA-dependent ATP production (*SI Appendix*, Fig. S7). This is in line with the earlier observation that the poor Pcd-kinetics, and thus lower electron transfer through the ETC, likely allows electrons to escape to oxygen before reaching the final coupling enzyme (Fig. 1*E*). Nevertheless, these findings show that direct coupling of artificial respiratory chains to efficient oxidation reactions, such as the Mcd-catalyzed step, enables energy-conserving ATP regeneration in cell-free systems.

Closing the full CETCH cycle, we examined whether coupling the Mcd-driven artificial respiratory chain could regenerate ATP consumed during the cycle and thereby increase glycolate formation (Fig. 3*D*). To that end, we optimized the concentrations of PL and exogenously supplied ATP (*SI Appendix*, Fig. S8). At 1 mg mL^–1^ PLs and 1 mM ATP we observed 59 ± 2% conversion of ATP into glycolate with Mco, while conversion yield was increased to 73 ± 5% by the Mcd-based respiratory chain, corresponding to 142 ± 54 µM additional glycolate production (Fig. 3*D*). Surprisingly, glycolate formation was further stimulated when GramA was added, suggesting that, although the Mcd-dependent respiratory chain supports ATP conservation, dissipation of the proton motive force has other consequences under these conditions, where a high ATP concentration is present from the start. It is possible that GramA addition relieves thermodynamic constraints in Cyt*bo*_3_ catalysis and/or suppresses unfruitful hydrolysis of residual ATP through reverse ATPase reaction at high ATP starting concentrations. Nevertheless, in summary, these data showed that integrating artificial respiratory chains into metabolic cycles can be beneficial, but also highlighted the challenge of disentangling kinetic from thermodynamic contributions when these respiratory chains are directly integrated within metabolic cycles.

### Constructing a Multi-Input Respiratory Chain to Power Synthetic CO_2_ Fixation.

**Fig. 4.**
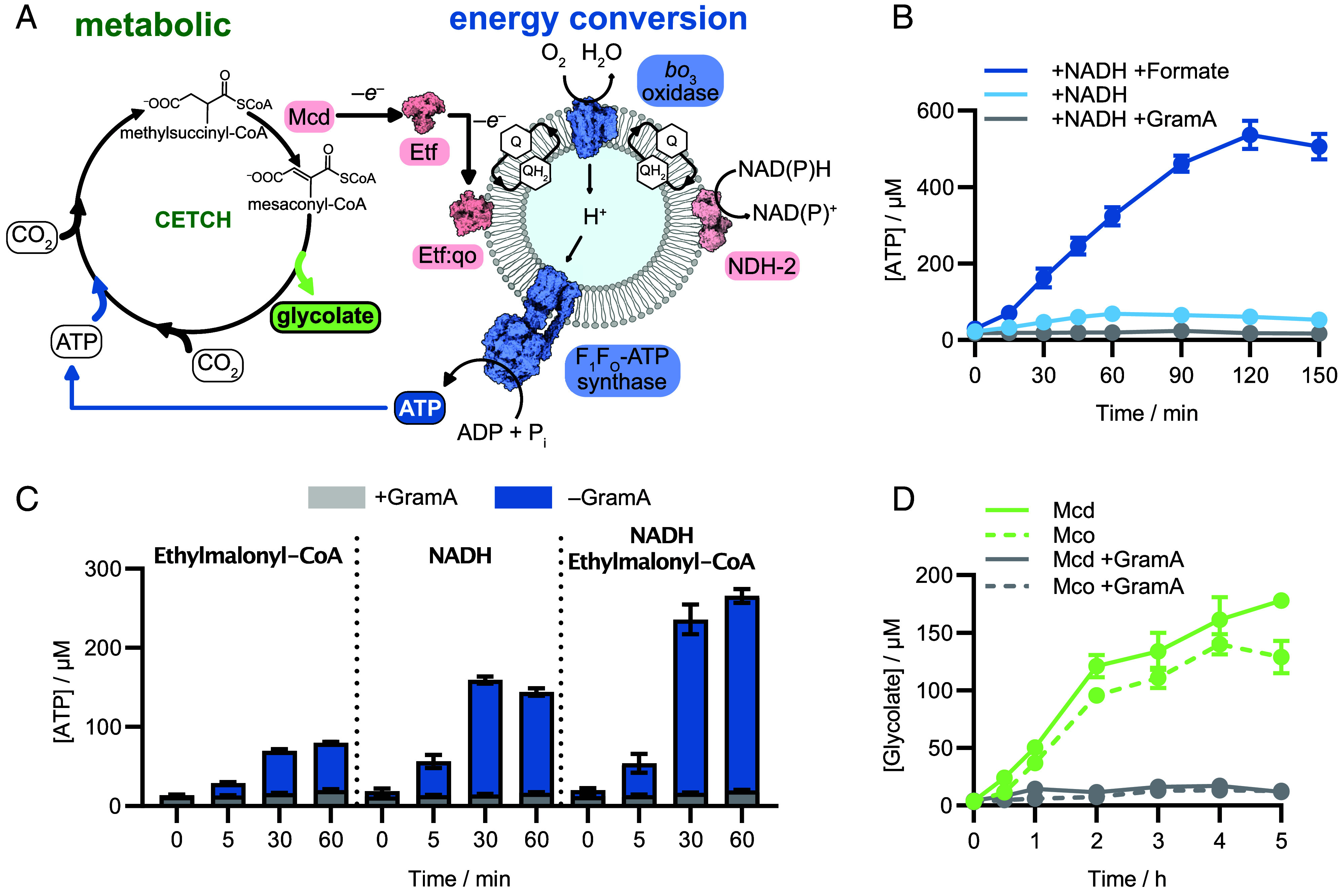
(*A*) Schematic of CETCH cycle powered by a multi-input artificial respiratory chain. The quinone pool is reduced by both the Etf-Etf:qo energy conserving electron transfer pathway, and the additional NDH-2-catalyzed NADH oxidation module. Regeneration of the quinone pool by *bo*_3_ oxidase generates a proton motive force to drive ATP synthesis, with the ATP produced available to sustain turnover of the CETCH cycle. (*B*) ATP synthesized from NDH2-Etf:qo-Cyt*bo*_3_-F_1_F_O_-PLs (3 mg mL^–1^) driven by the exogenous addition of NDH-2 (1 µM) and NADH (5 mM). ATP synthesis was abolished upon the addition of GramA (100 µg mL^–1^). Regeneration of the NADH-pool through formate (20 mM) and Fdh (30.6 µM) substantially increased ATP production. Data shown are the average of three technical replicates from four independent PL preparations ±SD. (*C*) ATP synthesized from NDH2-Etf:qo-Cyt*bo*_3_-F_1_F_O_-PLs (3 mg mL^–1^) in conditions where there are either one or two electron sources. ATP production was initiated by the addition of ethylmalonyl-CoA (5 mM) or NADH (5 mM), and GramA (100 µg mL^–1^) was added to uncouple the membranes. Ethylmalonyl-CoA was directly converted to methylsuccinyl-CoA by Epi and Ecm during the assay conditions. Data shown are the average of three technical replicates ±SD. The data are repeated in an independent PL preparation in *SI Appendix*, Fig. S11. (*D*) The complete CETCH cycle powered only by ATP generated from NDH2-Etf:qo-Cyt*bo*_3_-F_1_F_O_-PLs. All conditions contained NDH2-Etf:qo-Cyt*bo*_3_-F_1_F_O_-PLs (1 mg mL^–1^) but the methylsuccinyl-CoA to mesaconyl-CoA oxidation step were performed by either Mco with no Etf present, or with Mcd and Etf to connect the energy conservation module. The glycolate output was measured over 5 h after initiating with 100 µM propionyl-CoA. *SI Appendix*, Fig. S11 shows the effect of increasing the PL concentration to 3 mg mL^–1^ with an independent PL preparation under the same conditions. Data shown are the average of three technical replicates ±SD. and the concentration of all components are specified in *SI Appendix*, Table S2.

Drawing inspiration from natural respiration, we sought to introduce a second entry point for additional electrons to the UQ-pool to increase the ATP output, for which we aimed at employing NDH-2 catalyzed NADH (Fig. 4*A*). To provide NADH for NDH-2, we relied on an NADH/NADPH-promiscuous formate dehydrogenase (Fdh) that is already present as helper enzyme in the CETCH cycle (30). Interestingly, we observed that NADH-driven ATP production was substantially higher in PLs reconstituted with Etf:qo (*SI Appendix*, Fig. S4), and after further optimization (*SI Appendix*, Figs. S9 and S10), NDH-2-amended PLs were able to synthesize 537 ± 37 µM ATP after 2 h from 5 mM NADH (Fig. 4*B*). This is comparable to recent artificial ATP-generating proteoliposome platforms that employ alternative proton pumps, such as NADH oxidation at respiratory complex I (12), and the light driven activity of bacteriorhodopsin (11) and photosystem II (18). When testing with either ethylmalonyl-CoA or NADH as substrate, NDH2-Etf:qo-Cyt*bo*_3_-F_1_F_O_-PLs were able to synthesize ATP through methylsuccinyl-CoA oxidation (via Epi, Ecm, Mcd, Etf, Etf:qo) or NADH oxidation (via NDH-2), individually. Notably, ATP production was increased when both substrates were combined, indicating the parallel operation of both respiration routes (Fig. 4*C* and *SI Appendix*, Fig. S11).

Last, we integrated the complete CETCH cycle with NDH2-Etf:qo-Cyt*bo*_3_-F_1_F_O_-PLs demonstrating turnover of the CETCH cycle through glycolate production from concurrent ATP synthesis. Importantly, starting from only 100 µM propionyl-CoA, the yield of the cycle was increased by 38 ± 12% after 5 h in variants containing Mcd (and Etf) instead of Mco, indicating that direct integration of a tailored respiratory chain can strongly improve glycolate output (Fig. 4*D* and *SI Appendix*, Fig. S11). Yet, how much this improvement is the result of increased ATP generation from recursive coupling and/or improved kinetics of the respiratory chain remains difficult to distinguish.

### Linking Energy Metabolism and Information Processing.

Having established a tailored respiratory chain able to power metabolic networks, we became interested in the flexibility, robustness, and modularity of NDH2-Etf:qo-Cyt*bo*_3_-F_1_F_O_-PLs. We were especially interested on harnessing Fdh-based formate oxidation, because of the possibility to use this renewable organic acid (31–33) as a source for both reducing equivalents and energy. Beyond NADH, our respiration chain also showed activity toward NADPH, producing 29 ± 6% of the maximum ATP yield achieved with NADH after 1 h, due to an inherent promiscuity of NDH-2 (*SI Appendix*, Fig. S10). Moreover, our energy module retained approximately 88 ± 30% of its activity after 1 to 2 freeze–thaw cycles, demonstrating robustness for future applications (*SI Appendix*, Fig. S10).

**Fig. 5.**
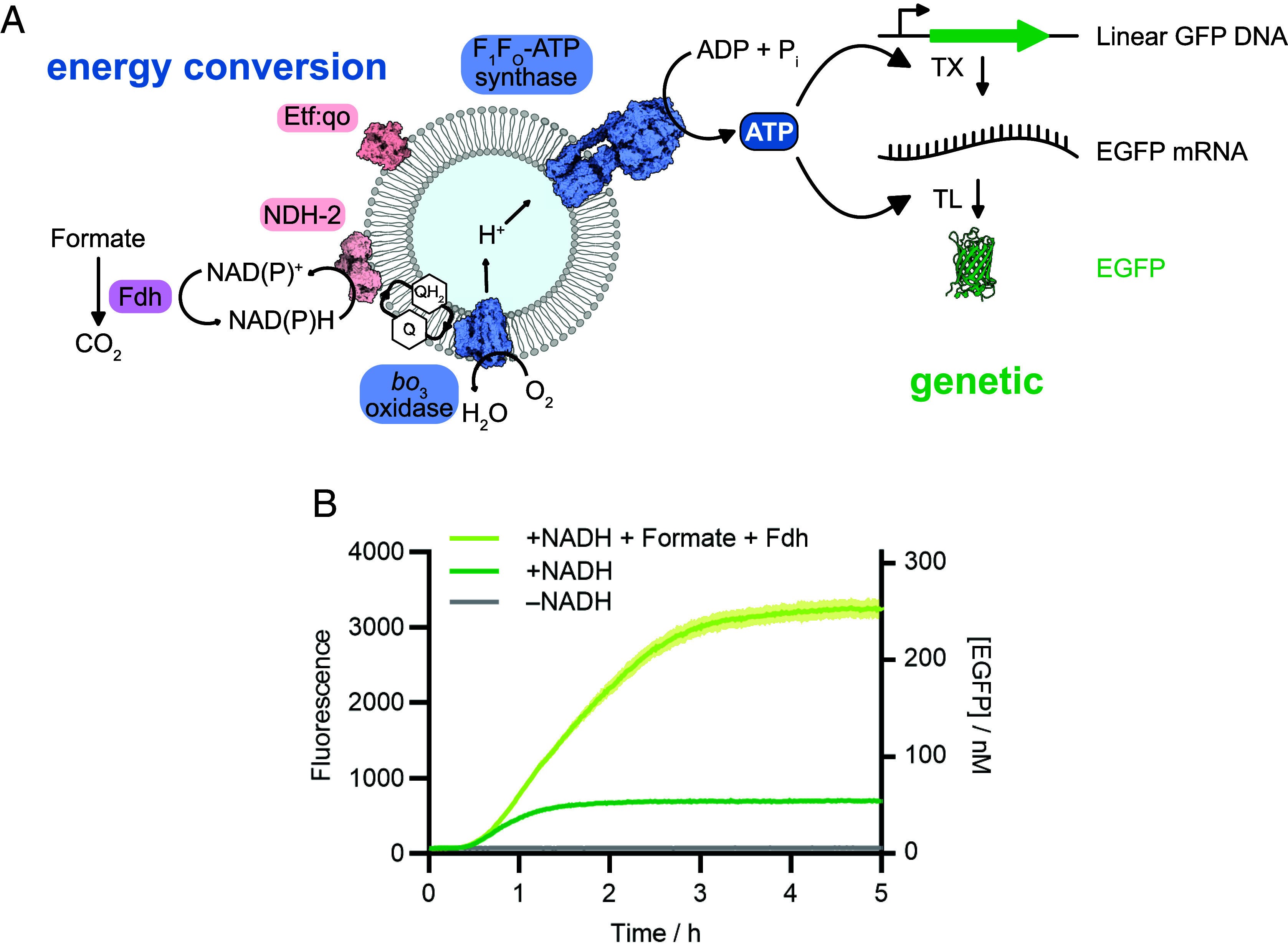
(*A*) Schematic showing the generation of EGFP in a modified PURE*frex* that is lacking ATP and ATP-regeneration machinery. Here, ATP is supplied by NDH2-Etf:qo-Cyt*bo*_3_-F_1_F_O_-PLs driven by NADH oxidation. Etf:qo was reconstituted into the PLs but did not take part in the ATP production. (*B*) EGFP output of NDH2-Etf:qo-Cyt*bo*_3_-F_1_F_O_-PLs (3 mg mL^–1^) in the presence of the modified PURE*frex* with and without formate (20 mM) and Fdh (30.6 µM) to regenerate the NAD(P)H (5 mM) pool. The EGFP concentration was calculated from a standard curve of purified EGFP measured under the same conditions (*SI Appendix*, Fig. S12). Data shown are the average of three technical replicates ±SD.

In respect to modularity, we sought to couple our respiratory chain with in vitro information processing, i.e., cell-free transcription–translation (TX-TL). To that end, we combined NDH2-Etf:qo-Cyt*bo*_3_-F_1_F_O_-PLs with a custom-prepared cell-free TX-TL system lacking ATP and ATP-regenerating enzymes (modified PURE*frex*). When provided with our energy module and formate, this PURE*frex* system was able to synthesize >280 nM enhanced green fluorescent protein (EGFP) with the kinetics of EGFP synthesis closely matching ATP production through our system (Figs. 4*B* and 5 and *SI Appendix*, Fig. S12). Overall, these experiments demonstrated another example of integrating biological functionalities, by successfully linking energy conversion with metabolism and information processing, paving the way toward the construction of more complex biological systems from the bottom–up.

## Discussion

With the ever growing bottom–up construction of complex reaction cascades and metabolic networks in synthetic biology, it becomes increasingly important to realize systems of higher functionality and integration. In this context, our study presents a step forward by constructing and tailoring artificial respiratory chains that facilitate the coupling of energy-conserving and metabolic processes.

By using the CETCH cycle as a model, we demonstrate how complex synthetic metabolic networks can be improved through such artificial respiratory chains. We show that these artificial respiratory chains provide kinetic advantages by facilitating rapid electron transfer steps, as well as thermodynamic benefits by concurrently conserving energy via synthesis of ATP. Thus, through implementing tailored energy modules within synthetic pathways the external energy dependencies of metabolic processes can be effectively reduced. We further demonstrate that additional entry points into artificial respiratory chains allow the use of multipurpose feed stocks, such as formate, which can provide both reducing power and additional energy (34), further simplifying and streamlining cell-free designs.

Our approach to construct tailored, membrane-based energy modules provides an alternative to well-established enzyme-based ATP-regeneration strategies that rely on externally added high energy (phosphorylated) compounds (35, 36), or more innovative designs, such as electrobiological ATP regeneration (7) that require anoxic conditions and are only of limited biocompatibility, as well as formate based membrane-independent enzyme cascades (34). Compared to the latter, our liposome-based system offers the additional advantage to expand metabolic networks by including membrane-based biochemistries and/or compartmentalizing noncompatible enzyme reactions in the future. Past and ongoing research aimed at enhancing the functionality, efficiency, and stability of proteoliposomes and other synthetic membranes, will contribute to the development of more robust complex systems (15), including improving membrane integrity and orientation of membrane proteins (9, 37–41). This might become especially important for *bo*_3_ oxidase, which has been shown to preferentially orientate inward facing in negatively charged liposomes, opposing proton motive force generation inside the vesicle lumen (41). Our results further underscore that maintaining a high ATP/ADP ratio is necessary to sustain adequate metabolic flux, and that improving the currently low ATP-synthesis efficiency of liposome-based systems will be critical to achieving this in the future.

Our artificial respiratory chains mimic natural energy-conserving process and thus introduces a critical component for achieving life-like functionalities and complexities that are self-sustaining. This framework serves as a promising foundation for the development of synthetic life-like systems endowed with novel features, ultimately bridging the gap between synthetic biology and the principles of life.

## Methods

### Production and Purification of Recombinant Proteins.

Enzymes were produced in *E. coli* strains BL21(DE3) and BL21 DE3 ArcticExpress (DE3)RIL unless otherwise stated. Cells were grown in terrific broth (TB) medium at 37 ˚C with shaking at 140 rpm using the appropriate antibiotic for selection (100 μg mL^–1^ ampicillin, 50 μg mL^–1^ kanamycin, 34 μg mL^–1^ chloramphenicol, 20 μg mL^–1^ streptomycin, 10 μg mL^–1^ tetracycline, and 50 μg mL^–1^ spectinomycin). Unless otherwise noted, when an OD_600_ = 0.6 to 1.0 was reached, the culture was cooled to 23 ˚C and induced by the addition of 500 μM IPTG (isopropyl-D-βthiogalactopyranoside). After 14 to 18 h of shaking at 140 rpm, the cells were harvested for 15 min at 5,000 × g at 4 ˚C. Pellets were resuspended at a 1:2 (w/v) ratio in buffer [50 mM HEPES pH 7.8, 500 mM NaCl, 5 mM MgCl_2_, 10% (v/v) glycerol, and DNase I] and lysed by ultrasonication using a KE76 probe (1 s on/off for total of 1 min on). The lysate was clarified by ultracentrifugation (45 min. at 100,000 × g) and the resulting supernatant was filtered through a 0.45 µm filter. Lysate was loaded onto a HisTrap FF (Cytiva) and nonspecific protein removed using 50 mM HEPES pH 7.8, 500 mM NaCl, 5 mM MgCl_2_, 10% (v/v) glycerol, and 50 to 75 mM imidazole. The protein was eluted in 50 mM HEPES pH 7.8, 500 mM NaCl, 5 mM MgCl_2_, 10% (v/v) glycerol and 500 mM imidazole. The eluted protein was either desalted in a buffer containing: 50 mM HEPES pH 7.8, 200 mM NaCl, 5 mM MgCl_2_, 10% (v/v) glycerol with a 2 × 5 mL HiTrap desalting column (Cytiva) or by gel filtration using a HiLoad 16/600 Superdex 200 pg size exclusion column (GE Healthcare, Freiburg, Germany) equilibrated with the desalting buffer. Elution fractions were concentrated with Amicon Ultra-4 centrifugal filters (Merck Millipore). Glycerol [20 to 40% (v/v)] was added to purified proteins before being aliquoted, frozen in liquid N_2_ and stored at –70 ˚C. Protein concentration was determined using Pierce™ BCA Protein Assay Kits and using the molecular weight calculated on ProtParam. Enzyme purity was assessed by SDS–PAGE on 4 to 20% MP TGX gels (biorad) and imaged with GelStick imager (Intas) (*SI Appendix*, Fig. S13).

### Preparation of Membranes Proteins.

Colonies from *E. coli* were grown on TB medium, induced with IPTG, and harvested as described above unless otherwise stated. After homogenizing the cells in resuspension buffer (50 mM HEPES pH 7.5, 100 mM NaCl, 1 mM MgCl_2_, DNase I), the resuspension was lysed by passing three times through a LM10 Microfluidizer at 18,000 psi. Cell debris was removed by centrifugation at 20,000 × g for 30 min. and the membrane fraction was collected by ultracentrifugation at 100,000 × g for 2 h. Membranes were suspended in (50 mM HEPES pH 7.5, 100 mM NaCl, 1 mM MgCl_2_) and flash frozen in liquid N_2_.

### Production and Purification of Etf:qo.

Etf:qo from *C. sphaeroides* was produced in *E. coli* BL21 (DE3) Suf^++^ cells (42) from a codon optimized pET28 plasmid with an N-terminal hexahistidine tag (*SI Appendix*, Table S3). Production and induction conditions were the same as above but the TB medium was supplemented with 5 mg of riboflavin and 0.5 mg of ferric citrate. Membranes were prepared as above. To solubilize the protein, membranes were diluted to 20 mg mL^–1^ and incubated while stirring at 4 ˚C with 2% (w/v) dodecyl-β-D-maltoside (DDM). Purification then proceeded as above but ensuring all purification buffers were supplemented with 0.05% (w/v) DDM. Protein was frozen in liquid N_2_ and stored at –70 ˚C.

### Production and Purification of NDH-2.

Alternative NADH dehydrogenase (NDH-2) was produced in *E. coli* BL21 (DE3) cells from a pET28 plasmid with a C-terminal hexahistidine tag (*SI Appendix*, Table S3). Cells were grown and protein production induced as above in TB medium supplemented with 5 mg of riboflavin. To solubilize the protein, membranes were diluted to 10 mg mL^–1^ and incubated while stirring at 4 ˚C with 2% (w/v) DDM. Purification then proceeded as above but ensuring all purification buffers were supplemented with 0.05% (w/v) DDM. Protein was frozen in liquid N_2_ and stored at –70 ˚C.

### Production and Purification of AOX.

AOX from *Trypanosoma brucei brucei* containing a N-terminal Twin-strep tag was recombinantly produced in *E. coli* BL21 (DE3) cell and purified according to Fedor and Hirst (26). Briefly, a cell were grown in 250 mL medium at 37 ˚C, 180 rpm containing 10 g L^–1^ tryptone, 5 g L^–1^ yeast extract, 5 g L^–1^ casamino acids, 10.4 g L^–1^ K_2_HPO_4_, 3 g L^–1^ KH_2_PO_4_, 0.74 g L^–1^ trisodium citrate dihydrate, 2.5 g L^–1^ (NH_4_)_2_SO_4_, 100 mg L^–1^ ampicillin, 50 mg L^–1^ kanamycin, 0.05 g L^–1^ MgSO_4_, 0.025 g L^–1^ FeSO_4_, 0.025 g L^–1^ FeCl_3_, 0.2% (w/v) glucose, 3 mL antifoam 204 until OD_600_ = 0.8. This was used to inoculate 6 × 1 L of the above medium and cells were grown under the same conditions until mid-exponential (OD_600_ ~0.7). Protein production was induced with 25 µM IPTG and cells grown overnight at 22 ˚C, 180 rpm. Cells were then harvested and membranes isolated using the standard protocol above using buffer containing 50 mM Tris-SO_4_ pH 7.5, 1 mM MgSO_4_, and few flakes of DNase I. To purify, membranes (6 mg mL^–1^ protein content) were solubilized for 30 min at 4 ˚C with stirring in buffer containing 25 mM Tris-HCl pH 7.5, 200 mM MgSO_4_, 20% (v/v) glycerol and 1.4% (w/v) n-Octyl β-D-glucopyranoside (OG). Insoluble material was removed by centrifugation at 100,000 × g for 45 min and the supernatant loaded onto a 5 mL Strep-Tactin Superflow (IBA Lifesciences) equilibrated in wash buffer containing 20 mM Tris-HCl pH 7.5, 50 mM MgSO_4_, 160 mM NaCl 20% (v/v) glycerol and 0.042% (w/v) DDM. Protein was eluted with 2.5 mM desthiobiotin and the sample dialyzed overnight into the wash buffer. Protein was aliquoted, frozen in liquid N_2_ and stored at –70 ˚C.

### Production and Purification of Cytochrome *bo*_3_ Oxidase.

Cytochrome *bo*_3_ oxidase from *E. coli* was purified as described previously (8, 43). The pETcyo plasmid encoding the *bo*_3_ operon with a C-terminal octahistidine tag on the cyoA subunit was transformed into BL21 (ΔcyoABCDE) *E. coli* cells and grown in supplemented M63 minimal medium [15 mM (NH_4_)_2_SO_4_, 100 mM KH_2_PO_4_ pH 7.0, 0.2% (v/v) glycerol, 1 mM MgSO_4_, 10 µM CuSO_4_, 1 µM FeSO_4_, 10 mg mL^–1^ thiamine, 100 mg mL^–1^ glycine and 0.001% (v/v) antifoam] at 37 ˚C, 160 rpm shaking until mid-exponential (OD_600_ = 0.6 to 0.8) where protein production was induced with 0.5 mM IPTG. Cells were incubated for 16 h at 20 ˚C, 160 rpm shaking. Membranes were prepared with the standard protocol above, followed by solubilization of the membranes (10 mg mL^–1^) with 2% (w/v) DDM. The insoluble fraction was removed after centrifugation at 100,000 × g for 45 min and the supernatant loaded onto a 2 × 5 mL HisTrap (Cytiva) pre-equilibrated in buffer [50 mM HEPES pH 7.5, 20 mM NaCl, 0.05% (w/v) DDM]. The column was washed with 75 mM imidazole and protein eluted with 500 mM imidazole. Protein was desalted on 2 × 5 mL HiTrap (Cytiva), concentrated, 20% (v/v) glycerol added, and frozen in liquid nitrogen in aliquots.

### Production and Purification of F_1_F_O_-ATP Synthase.

F_1_F_O_-ATP synthase from *E. coli* was purified as described previously (12, 44). The pBWU13 plasmid, encoding the entire *atp* operon with a His_6_-tag on the N-terminus of the β subunits, was transformed into *E. coli* DK8 cells, where the atp operon was deleted. Cells were grown in LB medium (60 mL) containing ampicillin (100 µg mL^–1^) and tetracycline (20 µg mL^–1^) at 37 ˚C, 200 rpm shaking until mid-exponential. Then TB medium (3 × 2 L), supplemented with MgCl_2_ (1 mM) and ampicillin (100 µg mL^–1^), was inoculated with 20 mL of preculture per flask and the cells were grown for 16 h at 37 ˚C, 220 rpm shaking. Cells were harvested and membranes isolated as above but using resuspension buffer containing 50 mM HEPES pH 8.0, 100 mM MgCl_2_, 100 mM NaCl, and 5% (v/v) glycerol. To purify, lauryl maltose neopentyl glycol (LMNG) [2% (w/v)] was added dropwise to membranes (30 mg mL^–1^) with constant stirring on ice for 30 min. Buffer [50 mM HEPES pH 8.0, 100 mM NaCl, 5 mM MgCl_2_, 30 g L^–1^ sucrose, 10% (v/v) glycerol] was added to dilute the membranes to 15 mg mL^–1^ and stirring continued for 30 min on ice. The insoluble fraction was removed after centrifugation at 50,000 × g for 45 min and the supernatant loaded onto a 5 mL HisTrap (Cytiva) pre-equilibrated in the same buffer plus 0.005% (w/v) LMNG. The column was washed with 75 mM imidazole and protein eluted with 500 mM imidazole. Protein was concentrated and frozen in liquid nitrogen in aliquots.

### Liposomes Preparation and Protein Reconstitution.

Proteoliposomes were prepared using a protocol adapted from Biner et al (12). For a standard reconstitution, 10 mg of synthetic lipids were mixed together (from Avanti Research) as 25 mg mL^−1^ stocks in chloroform and the chloroform evaporated off under nitrogen, followed by drying under vacuum for 1 h. The lipid mixes were prepared at a ratio of either 26:42:22:10 [% (w/w)] dioleoyl phosphocholine (DOPC): dioleoyl phosphoethanolamine (DOPE): dioleoyl phosphocholine (DOPG): cardiolipin (CDL), or 60:20:10:10 [% (w/w)] DOPC:DOPG:DOPE:CDL for respective preparations resembling the *C. sphaeroides* lipid composition or all other liposome preparations, unless otherwise stated. Ubiquinone-10 (150 nmol) from a 6.6 mM stock in chloroform was also mixed and dried. After drying, the lipids were hydrated in 1 mL of proteoliposome buffer (10 mM HEPES pH 7.8, 50 mM KCl) for 30 min with frequent vortexing and then extruded 11 times through a 100 nm Nucleopore polycarbonate membrane (Whatman). For reconstitution of F_1_F_O_-ATP synthase, *bo*_3_ oxidase and Etf:qo, 400 µL liposomes (10 mg mL^–1^) were partially solubilized by addition of 0.5% (w/v) sodium cholate [from 20% (w/v) stock] and the solution was incubated on ice for 10 min after mixing briefly by inversion. Then, 207 µg F_1_F_O_-ATP synthase, 35 µg *bo*_3_ oxidase and 119 µg Etf:qo, were added in that order where applicable. Reconstitution buffer were added to a final volume of 500 µL and the solution mixed by inversion and incubated at 4 ˚C for 15 min. Cholate was then removed using a PD10 desalting column (Cytiva) at 4 °C. PLs were collected by centrifugation at 100,000 × g (4 ˚C for 1 h), and resuspended in 200 μL ice-cold reconstitution buffer (~20 mg mL^–1^ final lipid concentration), and stored at 4 ˚C.

### Synthesis and Purification of acyl-CoA.

#### Propionyl-CoA and Crotonyl-CoA synthesis.

Propionic and crotonic, anhydride were used to synthesize their respective acyl-CoA using the symmetric anhydride method (27). First, coenzyme A (60 mg) was dissolved in 500 mM KHCO_3_ (4 mL) on ice. An approximately 4 × molar excess of anhydride was added, and the solution incubated on ice with constant agitation for 1 h. Reaction completion was confirmed with Ellman’s reagent.

#### Ethylmalonyl-CoA synthesis.

Ethylmalonyl-CoA was synthesized enzymatically from crotonyl-CoA. Crotonyl-CoA (15 mg) was dissolved in buffer (4 mL) containing 100 mM HEPES pH 7.8, 100 mM KHCO_3_, NADPH (24 mM), and 0.07 µM carbonic anhydrase. Ccr (2 µM) was added and reaction mixture incubated at 30 ˚C with shaking at 150 rpm for 1 h. Reaction was quenched in 10% (v/v) formic acid and precipitate removed after centrifugation at 4,500 × g for 15 min and filtering through 0.45 µm membrane.

#### CoA ester purification.

CoA esters were purified with a Gemini 10 μm NX-C18 110 Å Column (Phenomenex, Aschaffenburg, Germany) in a 1,260 Infinity HPLC (Agilent Technologies GmbH) using a methanol/sodium formate (pH 4.2) gradient. Purified fractions were frozen in liquid nitrogen, lyophilized, and stored with desiccant at 20 ˚C. CoA-thioester concentration was determined by spectrophotometric absorbance at 260 nm with ε = 16.4 mM^−1^ cm^−1^ for saturated CoA-thioesters.

### Biochemical Assays and Quantification of Reaction Products.

#### CETCH assays.

CETCH assay conditions were based on those previously optimized (20). CETCH (75 mM HEPES buffer pH 7.8, 12.5 mM MgCl_2_, 60 mM creatine phosphate, 2.5 mM potassium bicarbonate, 20 mM sodium formate, 0.4 mM CoA, 3 mM ATP, 3.75 mM NADPH, 1.9 μM Ccr, 0.7 μM Epi, 1.4 µM Ecm, 26 µM Mco, 0.3 µM Mch, 3.6 µM Mcl-1, 5 µM Gor, 3.1 µM Pco, 2.9 µM Mcm, 3.5 µM Scr, 1.7 µM Ssr, 0.5 µM Hbs, 0.7 µM Hbd, 3.3 µM Cat, 30.6 µM Fdh, 0.8 µM CK, and 70 nM Ca. For assays without Mco, 4 µM Mcd, 4 µM Etf, 4 µM Etf:qo, 0.26 µM AOX, and 1 mg (lipids) mL^–1^ PLs were generally used. For conditions with energy regeneration from PLs, the ATP, creatine kinase, and creatine phosphate were excluded from the reaction and 2 mM ADP, 10 mM KPO_4_, and 3 mg (lipids) mL^–1^ Etf:qo-Cyt*bo*_3_-F_1_F_O_-PLs were usually added unless otherwise stated. Reactions were initiated with propionyl-CoA (100 µM) and incubated up to 4 h at 32 ˚C with shaking at 800 rpm in a thermoshaker. Samples (10 µL) were quenched in 50% (v/v) formic acid (1 µL) and centrifuged at 1,739 × g for 30 min before diluting 10-fold in water. Samples were mixed 1:1 (v/v) sample:internal standard (20 μM ^13^C_2_-glycolate) prior to mass spectrometry analysis to account for matrix effects.

### ATP Synthesis Assays.

For ATP synthesis assays, reactions contained 75 mM HEPES pH 7.8, 12.5 mM MgCl_2_, 2 mM ADP, 10 mM KPO_4_, and 3 mg mL^–1^ PLs. Additional enzymes and supplements were added as described in *SI Appendix*. Reactions were initiated with either 5 mM ethylmalonyl-CoA, 5 mM crotonyl-CoA, 5 mM propionyl-CoA, or 5 mM NAD(P)H. Samples were incubated at 32 ˚C with shaking at 800 rpm in a thermoshaker. To uncouple the membrane under certain conditions, gramicidin A was used at a concentration of 100 µg mL^–1^ (Sigma Aldrich, Cat. No. 368020). In a 384-well, white, flat-bottom luminescence plate, samples (2 µL) were quenched into 4% (v/v) trifluoracetic acid (8 µL), followed by addition of 1 M Tris-HCl pH 8.0 (80 µL) 10 s later. Then 10 µL of the Roche ATP Bioluminescence Assay Kit CLS-II kit reagent was added to each well and the total luminescence of each well was measured using an Infinite M plex microplate reader (Tecan) with 1 s integration time. ATP concentrations were calculated by comparison to a standard curve generated alongside from samples of known ATP concentrations.

### Oxygen Consumption Assays.

The consumption of oxygen for the CETCH oxidation reactions were measured using a PyroScience fiber optic oxygen probe with an optically isolated minisensor (OXF1100-OI). The reactions were performed in a volume of 30 µL at 32 ˚C in buffer containing 50 mM HEPES pH 7.8, 10 mM MgCl_2_, and 50 mM KCl. For the measurement of the Mco/Mcd catalyzed oxidation step, reactions were initiated with ethylmalonyl-CoA (100 µM). Epi (0.7 µM) and Ecm (1.4 µM) were kept at a constant concentration throughout all conditions to convert ethylmalonyl-CoA to methylsuccinyl-CoA in the assay. Enzymes and liposomes were included/excluded from the assay conditions at the following concentrations: Mco (10 µM), Mcd (2 µM), Etf (2 µM), Etf:qo (1 µM), AOX (0.26 µM), and liposomes (1 mg mL^–1^). For the measurement of the Pco/Pcd catalyzed oxidation step, reactions were initiated with propionyl-CoA (100 µM) and enzymes and liposomes were included/excluded from the assay conditions at the following concentrations: Pco (1 µM), Pcd (20 µM), Etf (2 µM), Etf:qo (1 µM), AOX (0.26 µM), and liposomes (1 mg mL^–1^). The background oxygen consumption activity without any of the ETC components present was subtracted from each.

### Cell-Free Transcription and Translation.

A 25 µL ATP free PURE TX-TL system was assembled as described in Luo and coworkers (7) by combining 3.4 µL solution A (–ATP- salts –tRNAs –AAs), 1.6 µL salts solution (180 mM Mg-acetate and 1.5 M K-glutamate), 2.5 µL tRNAs solution (560 A_260_ mL^–1^ tRNAs), 2.5 µL 20 proteinogenic AAs solution (3 mM each), 1.25 µL custom PURE*frex* 1.0 solution II Δ(MK, CK, NDK, PPiase), 1.25 µL PURE*frex* 1.0 solution III (ribosomes), 0.5 µL RNAse inhibitor, 75 ng EGFP template, 1 mM ADP, 10 mM KPO_4_, 3 mg mL^–1^ Cyt*bo*_3_-F_1_F_O_-PLs and 1 µM NDH-2. Formate (20 mM) and Fdh (30.6 µM) were added to relevant assay conditions. Solution A (-ATP-salts-tRNAs-AAs) contained 150 mM creatine phosphate, 0.15 mM folinic acid, 15 mM spermidine, 7.4 mM DTT, 14.7 mM GTP, 7.4 mM CTP, 7.4 mM UTP, and 370 mM HEPES pH 7.6 (45). EGFP template DNA was PCR amplified by using Phusion High-Fidelity DNA polymerase, EGFP encoding gBlock, forward primer and reverse primer (*SI Appendix*, *Supplementary note*). Reactions were initiated with either 0.5 µL NADH (10 mM) or buffer (for negative control), after adding nuclease-free water to bring the final volume to 25 µL. After gently mix, PURE TX-TL reactions were transferred into Nunc^TM^ 384-well black transparent bottom plate (Thermo Fisher Scientific), sealed with SealPlate film (Sigma-Aldrich), pulse centrifuged, and incubated at 32 ˚C in Infinite M plex microplate reader (Tecan) for 5 h. The plate reader parameters were the following: mode = fluorescence bottom reading, interval time = 1 min, λ_ex_ = 488 nm, λ_em_ = 515 nm, gain = 100, number of flashes = 25, integration time = 20 ms, shaking orbital duration = 20 s, and shaking orbital amplitude = 5.5 mm.

### HPLC-MS/MS Quantification.

#### Glycolate.

Quantitative determination of Glycolate was performed using a LC-MS/MS. The chromatographic separation was performed on an Agilent Infinity II 1290 HPLC system using a Kinetex EVO C18 column (150 × 1.7 mm, 3 μm particle size, 100 Å pore size, Phenomenex) connected to a guard column of similar specificity (20 × 2.1 mm, 5 μm particle size, Phenomenex) a constant flow rate of 0.1 mL min^–1^ with mobile phase A being 0.1% (v/v) formic acid in water and phase B being 0.1% (v/v) formic acid methanol (Honeywell, Morristown, NJ) at 25 ˚C. The injection volume was 1 µL.

The mobile phase profile consisted of the following steps and linear gradients: 0 to 4 min constant at 0% B; 4 to 6 min from 0 to 100% B; 6 to 7 min constant at 100% B; 7 to 7.1 min from 100 to 0% B; 7.1 to 12 min constant at 0% B. An Agilent 6470 mass spectrometer was used in negative mode with an electrospray ionization source and the following conditions: ESI spray voltage 4,500 V, nozzle voltage 500 V, sheath gas 300 °C at 11 L min^–1^, nebulizer pressure 45 psig and drying gas 90 ˚C at 11 L min^–1^. The target compound was identified based on its mass transitions and retention time compared to standards. Chromatograms were integrated using MassHunter software (Agilent, Santa Clara, CA). Absolute concentrations were calculated based on an external calibration curve prepared in fresh medium, and corrected for matrix effects and ion suppression using isotope dilution mass spectrometry (IDMS). Mass transitions, collision energies, dell accelerator voltages, and dwell times have been optimized using chemically pure standards. Parameter settings are given in *SI Appendix*, Table S4.

#### Glycolate and ATP.

Quantitative determination of Glycolate and ATP were performed using a LC-MS/MS. The chromatographic separation was performed on an Agilent Infinity II 1290 HPLC system using a SeQuant ZIC-pHILIC column (150 × 2.1 mm, 5 μm particle size, peek coated, Merck) connected to a guard column of similar specificity (20 × 2.1 mm, 5 μm particle size, Phenomenex) a constant flow rate of 0.1 mL min^–1^ with mobile phase A comprised of 10 mM ammonium acetate in water, pH 9, supplemented with medronic acid to a final concentration of 5 μM and mobile phase B being 10 mM ammonium acetate in 90:10 acetonitrile to water, pH 9, supplemented with medronic acid to a final concentration of 5 μM at 40 ˚C. The injection volume was 1 µL.

The mobile phase profile consisted of the following steps and linear gradients: 0 to 1 min constant at 75% B; 1 to 6 min from 75 to 40% B; 6 to 9 min constant at 40% B; 9 to 9.1 min from 40 to 75% B; 9.1 to 20 min constant at 75% B. An Agilent 6495 ion funnel mass spectrometer was used in negative ionization mode with an electrospray ionization source and the following conditions: ESI spray voltage 3,000 V, nozzle voltage 1,000 V, sheath gas 300 °C at 11 L min^–1^, nebulizer pressure 20 psig and drying gas 100 °C at 11 L min^–1^. Compounds were identified based on their mass transition and retention time compared to standards. Chromatograms were integrated using MassHunter software (Agilent, Santa Clara, CA). Absolute concentrations were determined based on an external standard curve. Mass transitions, collision energies, cell accelerator voltages, and dwell times have been optimized using chemically pure standards. Parameter settings of all targets are given in *SI Appendix*, Table S5.

## Supplementary Material

Appendix 01 (PDF)

## Data Availability

All study data are included in the article and/or *SI Appendix*.
