# Supplementary material for: Improving cell-free metabolism through direct integration of artificial respiratory chains
Source: Proc Natl Acad Sci U S A. 2026 Jul 2;123(27):e2613483123. doi: 10.1073/pnas.2613483123 (PMC13342846; doi:10.1073/pnas.2613483123)
Supplement: Supplementary file 1 — Appendix 01 (PDF) [file pnas.2613483123.sapp.pdf]

Supplementary information

Improving cell-free metabolism through direct integration of artificial respiratory chains

Owen D Jarman[1]*, Nitin Bohra[1,2], Peter Claus[3], Nicole Paczia[3], Tobias J Erb[1,4,5]*

[1]Max Planck Institute for Terrestrial Microbiology, Karl-von-Frisch-Str. 10, 35043 Marburg, Germany.

[2]Max Planck School Matter to Life, Heidelberg, Germany.

[3]Core Facility for Metabolomics and Small Molecule Mass Spectrometry, Max Planck Institute for Terrestrial Microbiology, Marburg, Germany.

[4]Center for Synthetic Microbiology (SYNMIKRO), Philipps University Marburg, Karl-von-Frisch-Str. 14, Marburg, Hessen 35043, Germany.

[5]Microbes-for-Climate (M4C) Cluster of Excellence, Synmikro, Marburg.

*Corresponding authors:

Email: toerb@mpi-marburg.mpg.de; owen.jarman@mpi-marbug.mpg.de

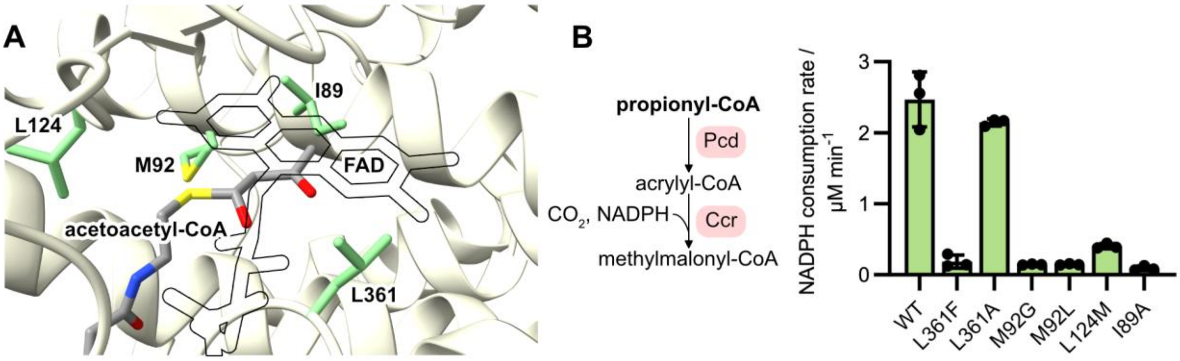

Figure S1. Active site residues mutated in Pcd and the activity of Pcd mutants for propionyl-CoA. (**A**) Alphafold structure of Pcd (Q3J1K6) overlayed with FAD and acetoacetyl-CoA from rat short chain acyl-CoA dehydrogenase (PDB: 1JQI). The residues mutated are shown in light green. Mutations chosen were designed to open or close the active site of Pcd in an attempt to change the preference from the putative native substrate (branched-chain acyl-CoA) to the linear propionyl-CoA. These include the mutations L124M and I89A. We also compared the active site of Pco (PDB: 2IX5) and replicated some of the different residues found in the active site. Specifically, residues M92 and L361 were substituted with a leucine and methionine at their respective positions in the Pco structure. All point mutations were created by site-directed mutagenesis on the Pcd-encoding plasmid and verified by sequencing. (**B**) Assay schematic and NADPH consumption rates for measuring activity of Pcd variants. The rate of NADPH consumption was measured spectroscopically at 340 nm in an Infinite M plex microplate reader (Tecan) as propionyl-CoA (100 µM) was converted to methylmalonyl-CoA through the Pcd and Ccr catalysed reaction steps. Assay buffer (50 mM HEPES pH 7.8, 10 mM $MgCl_2$) was supplemented with 2.5 mM $KHCO_3$, NADPH (100 µM), 1 mg mL^{-1} UQ_{10}-liposomes, Ccr (0.19 µM), Etf (1 µM), Etf:qo (1 µM), Pcd (5 µM), Cat (10 µM) and carbonic anhydrase (0.07 µM). Maximum initial rates are shown for three technical replicates ± S.D.

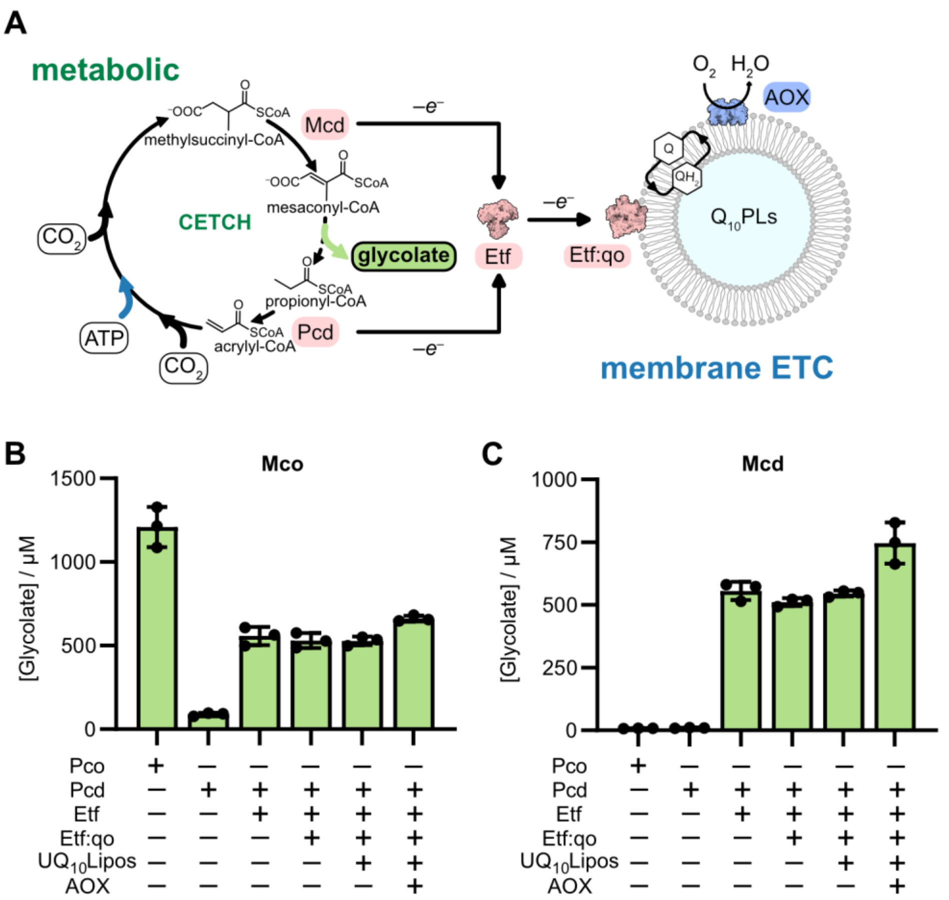

Figure S2. CETCH cycle turnover with propionyl-CoA dehydrogenase and methylsuccinyl-CoA dehydrogenase. (**A**) Schematic of the CETCH cycle with acyl-CoA oxidation steps transferring electrons through an artificial electron transport chain containing Etf, Etf:qo and AOX. (**B**) Glycolate produced from 100 µM propionyl-CoA when replacing Pco (3.1 µM) with Pcd (25 µM). The oxidation of methylsuccinyl-CoA was performed by Mco (26 µM) in these conditions. (**C**) Glycolate produced from 100 µM propionyl-CoA when replacing both oxidases (Mco (26 µM) and Pco (3.1 µM)) with equivalent dehydrogenases (Mcd (4 µM) and Pcd (25 µM)). ETC components were added at the following concentrations: Etf (4 µM), Etf:qo (4 µM), UQ_{10}-liposomes (3 mg mL^{-1}) and AOX (0.26 µM). In all cases, turnover was initiated upon addition of 100 µM propionyl-CoA and the glycolate produced was measured after 4 hours. Data shown are the average of three technical replicates ± S.D.

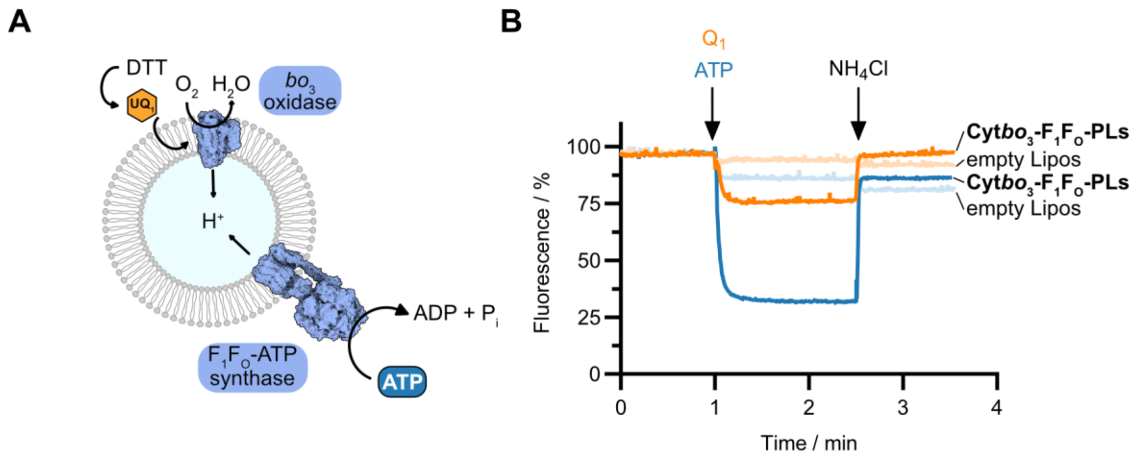

Figure S3. Assessing membrane coupling in Cyt*bo*$_3$-F$_1$F$_O$-PLs. (**A**) Scheme showing proton motive force generated by either *bo*$_3$ oxidase catalysis driven by DTT and UQ$_1$ reduction or F$_1$F$_O$-ATP synthase driven by ATP hydrolysis. (**B**) ACMA (9-amino-6-chloro-2-methoxyacridine) quenching of Cyt*bo*$_3$-F$_1$F$_O$-PLs and empty liposomes (0.4 mg mL^{-1} lipid concentration) showing the formation of a pH gradient (ΔpH). Fluorescence is normalised to the maximum fluorescence at the start of each measurement. Blue lines show formation of ΔpH from the F$_1$F$_O$ ATP-synthase working in reverse upon ATP addition (2 mM) and the orange line show ΔpH formation from Cyt*bo*$_3$ catalysis upon addition of UQ$_1$ (10 µM). The addition of NH_4Cl (10 mM) abolishes the pH gradient. Empty liposomes without *bo*$_3$ oxidase and F$_1$F$_O$-ATP synthase are shown for comparison. Assays were performed at 32 ˚C and with maximum stirring in Cary Eclipse Fluorescence Spectrophotometer (Agilent Technologies). ACMA fluorescence was measured every 0.25 s using excitation and emission wavelengths of 410 nm and 480 nm respectively. All conditions were recorded in assay buffer containing 50 mM HEPES pH 7.8,10 mM $MgCl_2$, 50 mM KCl, 2 mM DTT, 0.5 µM ACMA and 0.1 µM valinomycin. The addition of valinomycin dissipates the electrochemical membrane potential by transporting potassium ions out of the vesicle lumen and allowing for an increased pH gradient to be established. This increased the measurable fluorescence signals.

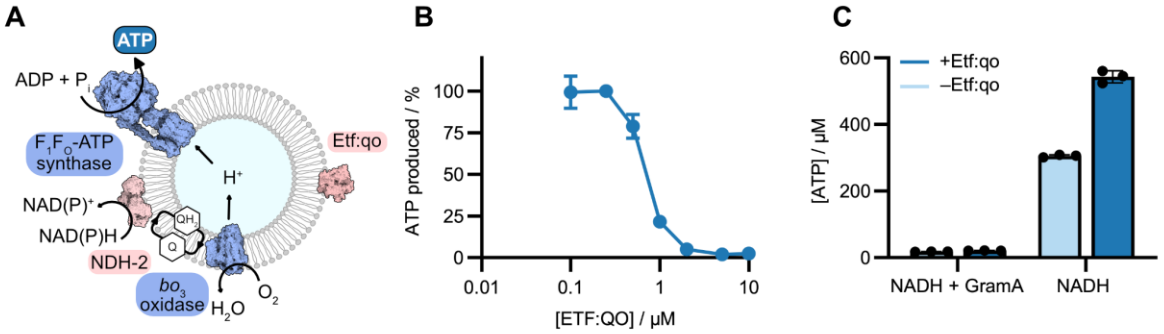

Figure S4. Effect of Etf:qo on Cyt*bo*$_3$-F_1F_O-PLs ATP synthesis (**A**) Schematic of the Cyt*bo*$_3$-F_1F_O-PLs synthesising ATP from NADH oxidation at NDH-2. Etf:qo is either titrated exogenously or reconstituted into these PLs in panels B and C respectively. (**B**) Relative percentage of ATP synthesised upon exogenous addition of Etf:qo into preformed Cyt*bo*$_3$-F_1F_O-PLs. The maximum amount of ATP synthesised through NADH oxidation with 1 µM NDH-2 was 445 µM. ATP synthesised dramatically drops off at >0.5 µM Etf:qo, which corresponds to a DDM concentration of ~0.0001% w/v. (**C**) Effect of Etf:qo reconstitution on NADH-driven ATP synthesis measured after 90 minutes. Synthesis was initiated by the addition of NADH (5 mM), and NADH was regenerated by Fdh (30.6 µM) and formate (20 mM). All assays contained 50 mM HEPES pH 7.8, 10 mM $MgCl_2$, 50 mM KCl, 2 mM ADP, 10 mM KPO_4, 3 mg mL^{-1} PLs and 1 µM NDH-2. Data shown are the average of three technical replicates ± S.D.

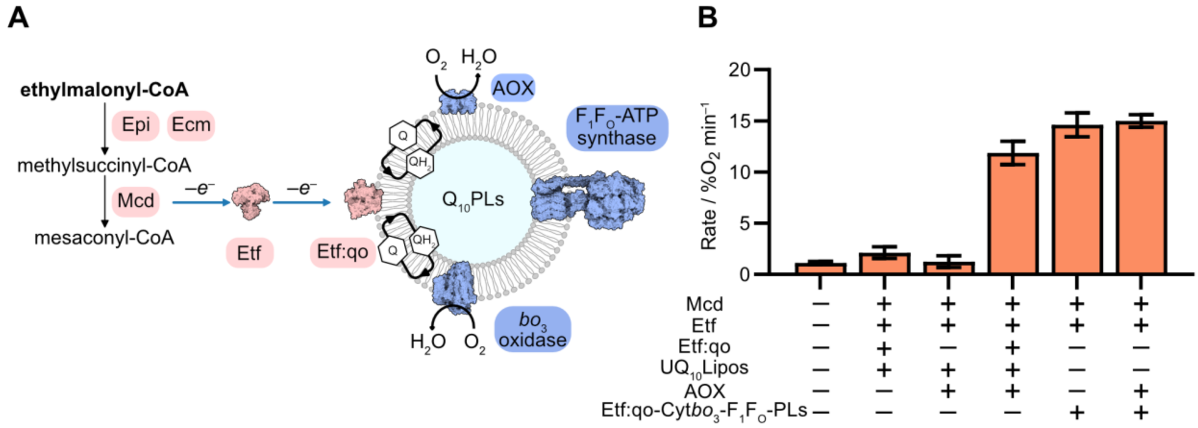

Figure S5. Comparison of the rate of oxygen consumption between different ETCs with AOX or *bo*$_3$ oxidase as the terminal quinol oxidase. (**A**) Schematic of oxygen consumption assay setup. Ubiquinone-10 is regenerated either through exogenous addition of AOX or through reconstitution of *bo*$_3$ oxidase into UQ$_{10}$-liposomes. (**B**) Initial oxygen consumption rates measured for different ETCs. All assays were performed in buffer containing 50 mM HEPES pH 7.8, 10 mM $MgCl_2$, 50 mM KCl, Epi (0.7 µM) and Ecm (1.4 µM). Each component of the ETC were added at the following concentrations: Mcd (2 µM), Etf (2 µM), Etf:qo (1 µM), AOX (0.26 µM), UQ$_{10}$Lipos (1 mg mL^{-1}) and Etf:qo-Cyt*bo*$_3$-F$_1$F$_O$-PLs (1 mg mL^{-1}). Assays were initiated by the addition of 100 µM ethylmalonyl-CoA. Oxygen consumption rates shown were not normalised to total ETC protein concentration as the protein concentration in the reconstituted Etf:qo-Cyt*bo*$_3$-F$_1$F$_O$-PLs was not measured and defined. Shown are the average of at least three individually measured replicates ± S.D.

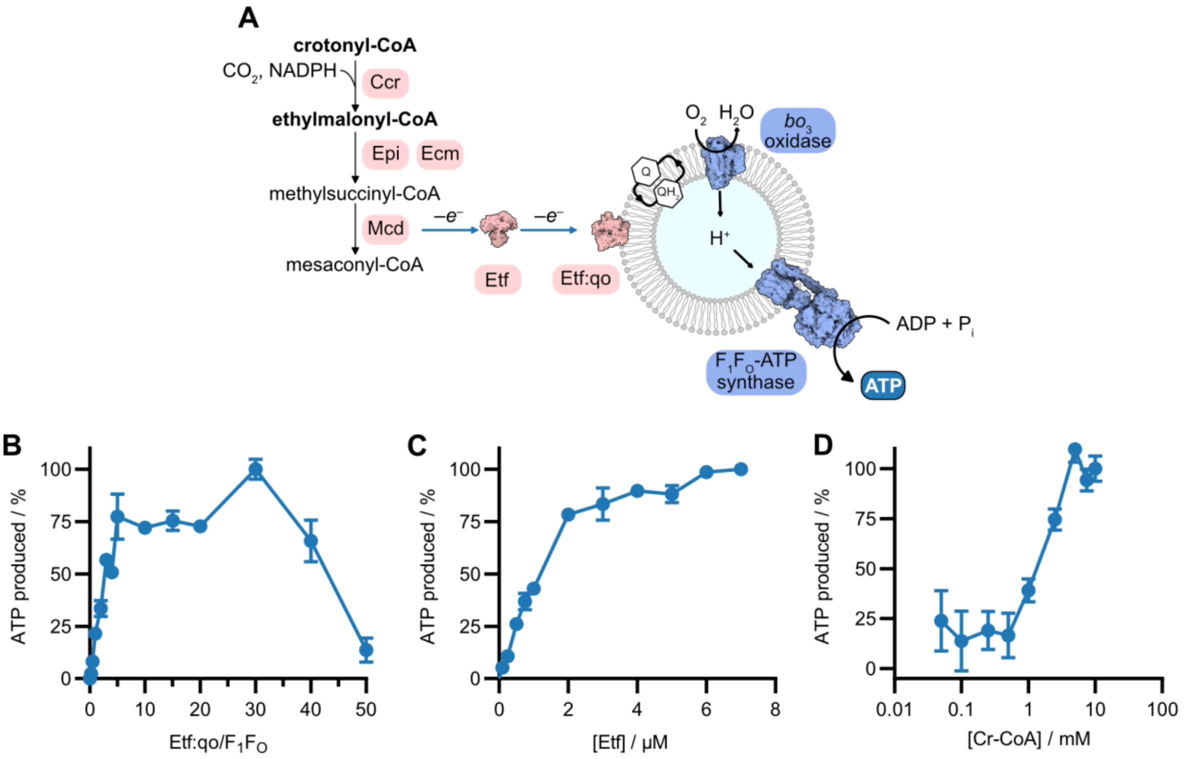

Figure S6. Optimisation of Etf:qo-Cyt*bo*$_3$-F$_1$F$_O$-PLs for acyl-CoA oxidation-driven ATP synthesis. (**A**) Schematic of Etf:qo-Cyt*bo*$_3$-F$_1$F$_O$-PLs supplied with electrons through methylsuccinyl-CoA dehydrogenase. Due to instability of the methylsuccinyl-CoA, ethylmalonyl-CoA was used to initiate activity for panels B and C. This was converted to methylsuccinyl-CoA by Epi (0.7 µM) and Ecm (1.4 µM) in the reaction mixture. (**B**) Effect of reconstitution ratio of Etf:qo to F$_1$F$_O$-ATP synthase on ATP synthesis through Mcd (3 µM). F$_1$F$_O$-ATP synthase and *bo*$_3$ oxidase were fixed at 9 µg and 52 µg per mg of lipid respectively and Etf:qo titrated relative to F$_1$F$_O$. A maximum of 60 µM ATP was synthesised after 1 hour upon addition of 2.8 mM ethylmalonyl-CoA. (**C**) Effect of Etf concentration on ATP synthesis in Etf:qo-Cyt*bo*$_3$-F$_1$F$_O$-PLs with 3 µM Mcd. Reaction was initiated with 5 mM ethylmalonyl-CoA and a maximum of 47 µM ATP was synthesised after 30 minutes. (**D**) Effect of crotonyl-CoA substrate concentration on ATP synthesis in Etf:qo-Cyt*bo*$_3$-F$_1$F$_O$-PLs. A maximum of 33 µM ATP was synthesised at a concentration of 5 mM crotonyl-CoA after 30 minutes. The reaction contained Ccr (1.9 µM), Epi (0.7 µM), Ecm (1.4 µM) Mcd (3 µM), Etf (3 uM), NADPH (3.75 mM), formate (3.75 mM), Fdh (30.6 µM), carbonic anhydrase (0.07 µM) and KHCO$_3$ (2.5 mM). All data shown are the average of three technical replicates ± S.D.

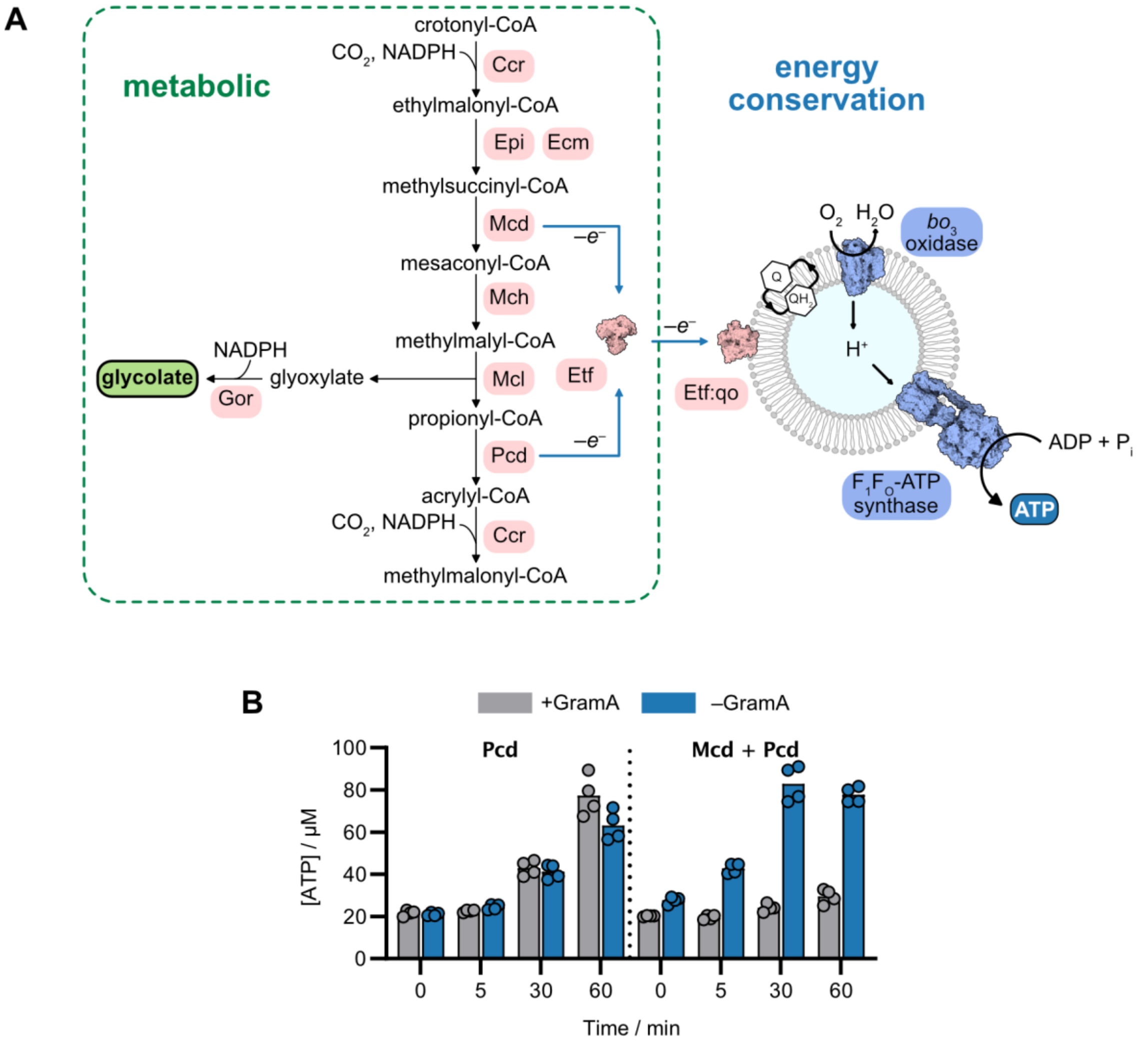

Figure S7. (**A**) Schematic of part of the CETCH cycle containing the two acyl-CoA oxidation reactions. Electrons from Mcd and Pcd catalysed reactions are shuttled through Etf to Etf:qo and the quinone pool is reoxidised through the proton-pumping *bo*$_3$ oxidase generating a proton motive force in the Etf:qo-Cyt*bo*$_3$-F_1F_O-PLs. (**B**) ATP synthesised from the Pcd-only or Mcd and Pcd-catalysed oxidation steps of the CETCH cycle conserved through an artificial respiratory chain (3 mg mL^{-1} Etf:qo-Cyt*bo*$_3$-F_1F_O-PLs). Catalysis was initiated with either propionyl-CoA (5 mM) or crotonyl-CoA (5 mM) to probe the ATP synthesised from Pcd-only or Mcd and Pcd-catalysed oxidation steps respectively. Experiments were performed in buffer containing 75 mM HEPES pH 7.8, 12.5 mM $MgCl_2$, 2 mM $KHCO_3$, 20 mM formate, 2 mM ADP, 10 mM KPO_4, 3.75 mM NADPH and 0.5 mM Ap5A. The enzymes were added at the following concentrations: Epi (0.7 µM), Ecm (1.4 µM), Mcd (3 µM), Mch (0.3 µM), Mcl-1 (3.6 µM), Gor (5 µM), Pcd (20 µM), Ca (0.07 µM), Fdh (30.6 µM) and Cat (3.3 µM). The protonophore gramicidin A (100 µg mL^{-1}) uncoupled the membranes showing any non-specific background ATP present in the assay setup. Data shown are the average of three technical replicates from four independent PL preparations ± S.D

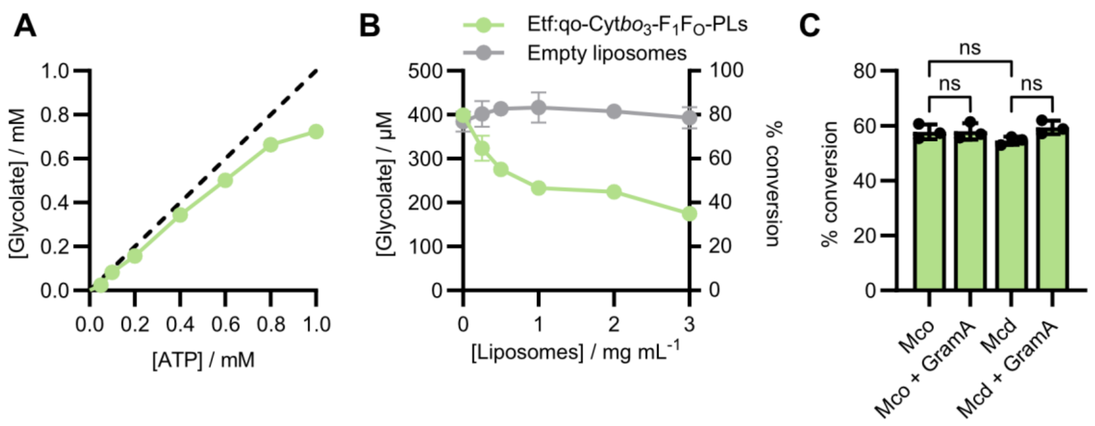

Figure S8. Glycolate produced versus known ATP quantities supplied to the CETCH cycle under various conditions. (**A**) Glycolate produced during 2 hours of CETCH cycle turnover upon addition of defined amounts of ATP. Dashed line shows theoretical maximum glycolate yield if all ATP is used. CETCH cycle was run in standard assay conditions containing Mco and Pco but without any ATP regeneration machinery (no creatine kinase/creatine phosphate or artificial respiratory chains (proteoliposomes)). The standard assay conditions can be seen in **Table S2, Figure 2**. (**B**) Glycolate produced after 4 hours of CETCH cycle turnover upon addition of 500 µM ATP in the presence of different concentrations of empty liposomes or Etf:qo-Cyt*bo*$_3$-F_1F_O-PLs. Turnover was initiated upon addition of ATP and propionyl-CoA (500 µM). Percentage conversion was calculated from the total glycolate yield divided by the starting ATP concentrations. The standard assay conditions for the CETCH cycle (Table S2, Figure 2) were used for all other component concentrations and Mco and Pco were used so the CETCH cycle turnover was not dependent on a membrane-bound ETC. Data shown are the average of three technical replicates ± S.D. The decrease in conversion upon Etf:qo-Cyt*bo*$_3$-F_1F_O-PLs addition suggest a negative interaction in the system, possibly due to ATP hydrolysis through increasing concentrations of the F_1F_O-ATPase in the system. (**C**) Conversion percentage of the complete CETCH cycle connected to the artificial respiratory chain (1 mg mL^{-1} Etf:qo-Cyt*bo*$_3$-F_1F_O-PLs) without any additional ATP regeneration machinery (creatine kinase and creatine phosphate). Turnover was initiated on addition of 0.5 mM propionyl-CoA and ATP and glycolate production measured after 4 hour incubation. Membranes were uncoupled with gramicidin A (100 µg mL^{-1}). Percentage conversion was calculated from the measured glycolate concentration and the starting ATP/propionyl-CoA concentration. Statistical significance was calculated by one-way ANOVA using Tukey's test, ns $p>0.5$. Data shown are the average of three technical replicates ± S.D. No significant change in the conversion percentage is seen between all conditions. The lower substrate concentration (500 µM ATP/propionyl-CoA) is likely insufficient to activate ATP synthesis, in agreement with substrate titration data in **Figure S6D**.

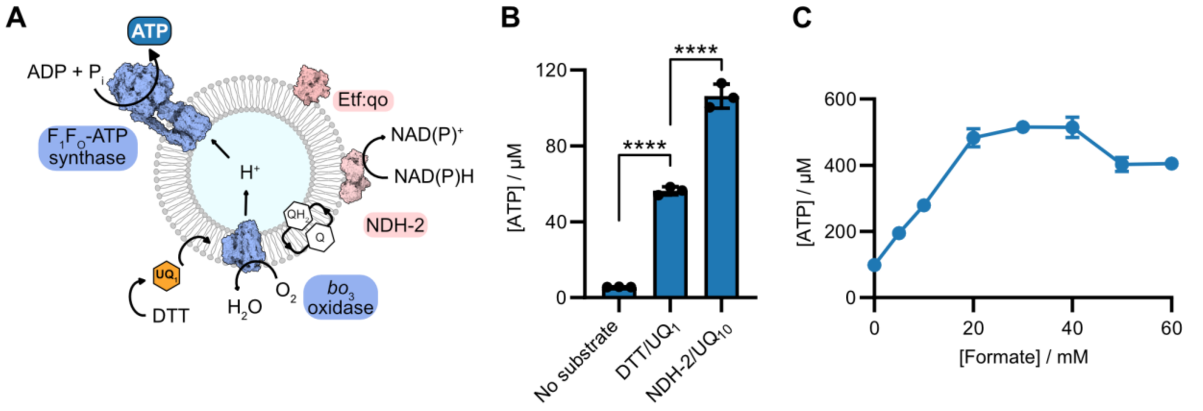

Figure S9. Comparison of ATP synthesis with short and long chain ubiquinone and NADH regeneration. (**A**) Scheme showing Etf:qo-Cyt*bo*$_3$-F$_1$F$_O$-PLs synthesising ATP from either UQ$_1$ or UQ$_{10}$. Etf:qo was reconstituted into the PLs but did not take part in the ATP production. (**B**) ATP synthesised after 1 hour at 32 ˚C for each condition. Either DTT (5 mM) and UQ$_1$ (10 µM), or NADH (5 mM) and NDH-2 (1 µM) were added to initiate ATP synthesis. Cyt*bo*$_3$-F$_1$F$_O$-PLs were prepared containing a concentration of 15 mM UQ$_{10}$ in the membrane. Data shown are the average of three technical replicates ± S.D. Statistical significance was calculated by one-way ANOVA using Tukey's test, ****$p<0.0001$ (**C**) Effect of formate concentration (and NADH regeneration) on total ATP synthesised after 1 hour. NDH2-Etf:qo-Cyt*bo*$_3$-F$_1$F$_O$-PLs were prepared and Fdh (30.6 µM) and NADH (5 mM) present in all conditions. Data shown are the average of three technical replicates ± S.D. Assay conditions in panels B and C contained 50 mM HEPES pH 7.8, 10 mM $MgCl_2$, 50 mM KCl, 2 mM ADP, 10 mM KPO_4 and 3 mg mL^{-1} PLs.

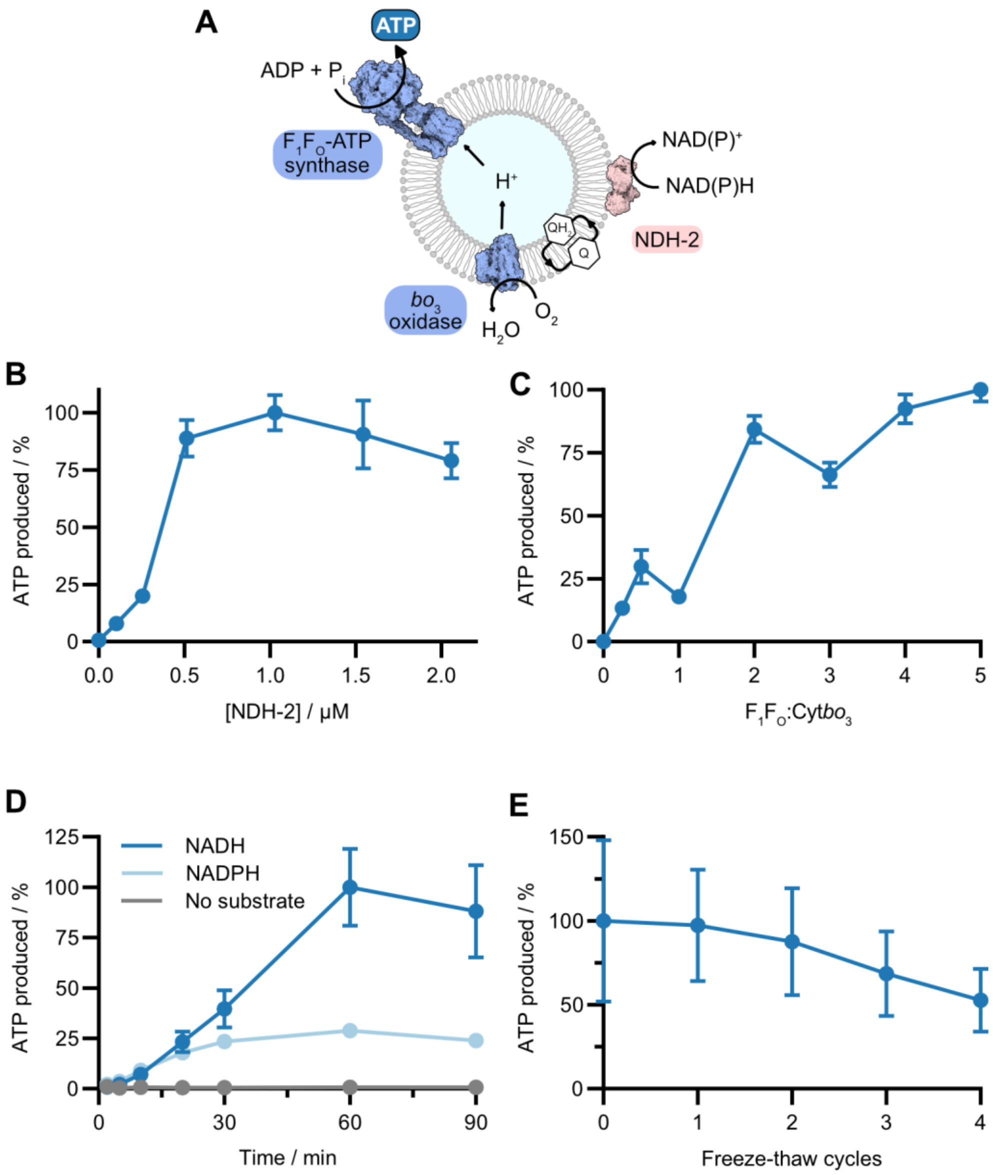

Figure S10. Optimisation of NDH2-Cyt*bo*$_3$-F$_1$F$_O$-PLs for ATP synthesis. (**A**) Schematic of NDH2-Cyt*bo*$_3$-F$_1$F$_O$-PLs synthesising ATP from NADH oxidation through NDH-2. This setup was used in all panels. Note the Etf:qo was not reconstituted into these liposomes (**B**) ATP synthesised after 1 hour at different NDH-2 concentrations added exogenously to Cyt*bo*$_3$-F$_1$F$_O$-PLs (3 mg mL^{-1}). A total of 214 µM ATP was synthesised at a concentration of 1 µM NDH-2 (**C**) Normalised ATP synthesised after 1 hour in Cyt*bo*$_3$-F$_1$F$_O$-PLs reconstituted with different ratios of Cyt*bo*$_3$:F$_1$F$_O$. The Cyt*bo*$_3$ concentration was fixed at 9 µg per mg of lipid (50 pmol per mg of lipid) and F$_1$F$_O$ titrated relative to this. A maximum of 54 µM ATP was synthesised at a ratio of 5:1 F$_1$F$_O$:Cyt*bo*$_3$. A ratio of 2:1 F$_1$F$_O$:Cyt*bo*$_3$ was used for all future experiments. (**D**) ATP synthesised over time for Cyt*bo*$_3$-F$_1$F$_O$-PLs (3 mg mL^{-1}) with either NADH (5 mM) or NADPH (5 mM) as the reductant. A maximum of 179 µM ATP was synthesised after 1 hour. (**E**) Normalised ATP synthesised after number of freeze-thaw cycles. Samples were flash frozen in liquid nitrogen followed by thawing at 25 ˚C. All values were

normalised to the maximum ATP yield (179 µM) without freezing. All assays were performed in buffer containing 50 mM HEPES pH 7.8, 10 mM $MgCl_2$, 50 mM KCl, 200 µM ADP, 10 mM KPO_4, 1 µM NDH-2 and 5 mM NADH. Data shown are the average of three technical replicates ± S.D.

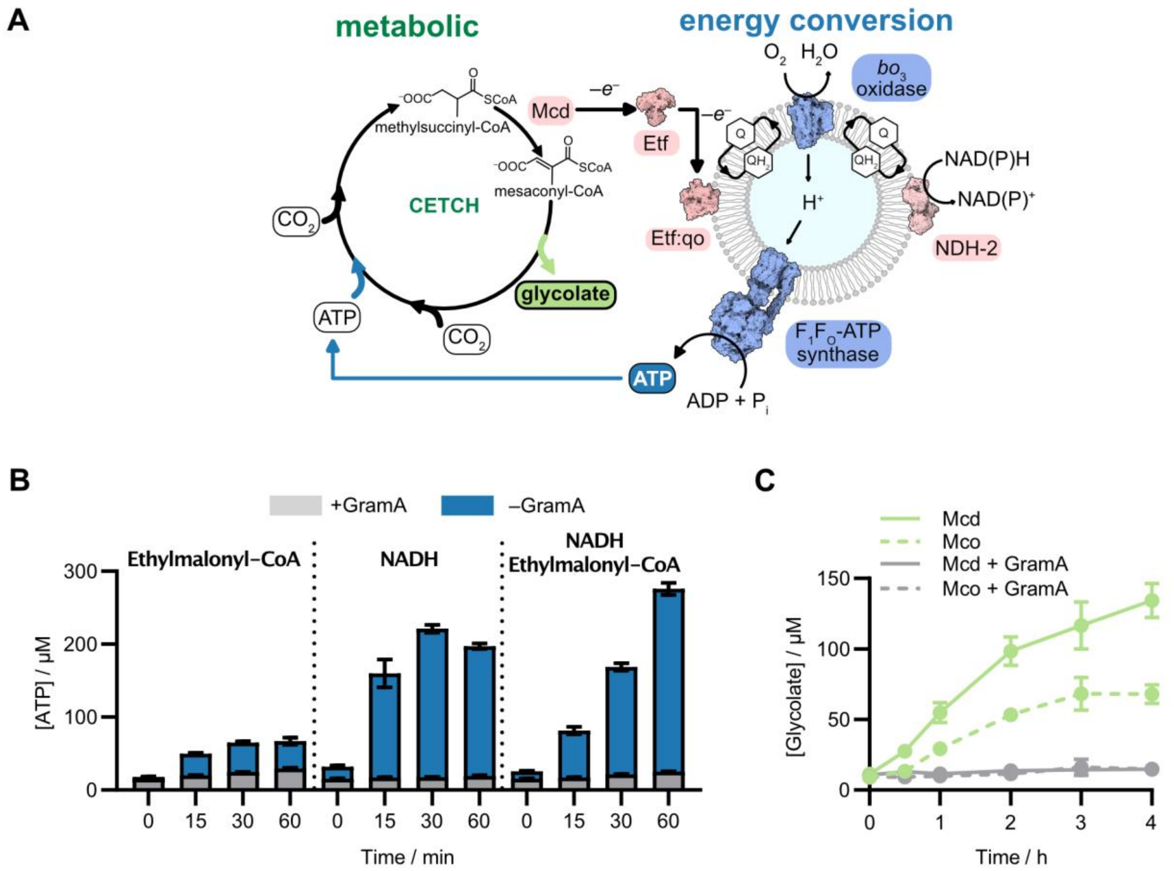

Figure S11. (**A**) Schematic of CETCH cycle powered by a multi-input artificial respiratory chain. The quinone pool is reduced by both the Etf-Etf:qo energy conserving electron transfer pathway, and the additional NDH-2-catalysed NADH oxidation module. Regeneration of the quinone pool by *bo*$_3$ oxidase generates a proton motive force to drive ATP synthesis, with the ATP produced available to sustain turnover of the CETCH cycle. (**B**) ATP synthesised from Etf:qo-Cyt*bo*$_3$-F_1F_O-PLs (3 mg mL^{-1}) in conditions where there are either one or two electron sources. ATP production was initiated by the addition of ethylmalonyl-CoA (5 mM) or NADH (5 mM), and GramA (100 µg mL^{-1}) was added to uncouple the membranes. All reaction conditions contained Epi (0.7 µM), Ecm (1.4 µM), Mcd (3 µM), Etf (3 µM), NDH-2 (1 µM), Fdh (30.6 µM) and 3.75 mM formate. Data shown are the average of three technical replicates ± S.D. The data is a repeated experiment of **Figure 3C** with an independent PL preparation. (**C**) The complete CETCH cycle powered only by ATP generated from Etf:qo-NDH2-Cyt*bo*$_3$-F_1F_O-PLs. All conditions contained Etf:qo-NDH2-Cyt*bo*$_3$-F_1F_O-PLs (3 mg mL^{-1}) but the methylsuccinyl-CoA to mesaconyl-CoA oxidation step were performed by either Mco (26 µM),

with no Etf present, or with Mcd (10 µM) and Etf (10 µM) to connect the energy conservation module. The glycolate output was measured after 4 hours after initiating with 100 µM propionyl-CoA. The concentration of all components are the same as specified in the conditions in **Table S2, Figure 4D**, apart from 3 mg mL^{-1} Etf:qo-NDH2-Cyt*bo*$_3$-F$_1$F$_O$-PLs were used. Data shown are the average of three technical replicates ± S.D.

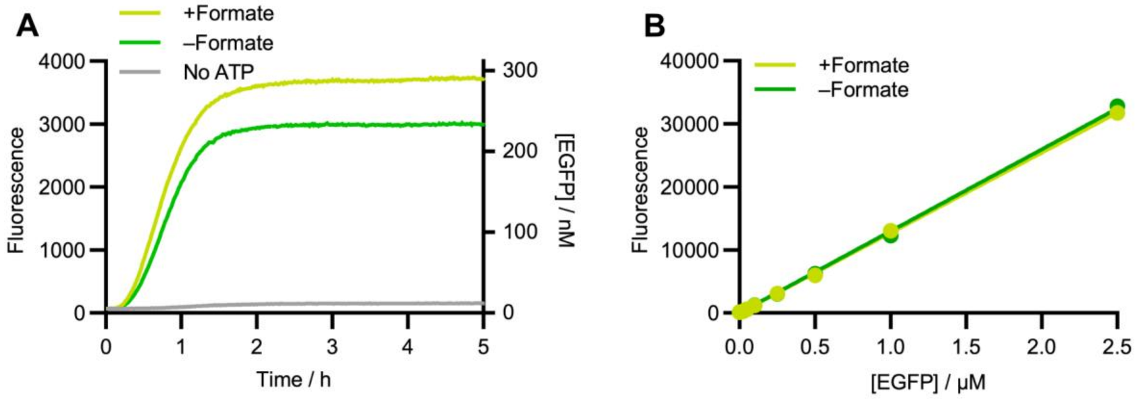

Figure S12. Production of EGFP from modified PURE*frex* containing no ATP regeneration machinery in the presence of NDH2-Etf:qo-Cyt*bo*$_3$-F$_1$F$_O$-PLs. (**A**) EGFP production in cell-free TX-TL when supplied with 500 µM ATP. All conditions contained NDH2-Etf:qo-Cyt*bo*$_3$-F$_1$F$_O$-PLs (3 mg mL^{-1}) but no NADH was present to drive ATP synthesis through the PLs. The amount of EGFP synthesised was calculated from the standard curve in panel B, and the effect of formate (20 mM) and Fdh (30.6 µM) on the production is shown. The grey line shows the fluorescence when no ATP was added to the system. (**B**) Standard curve of the fluorescence from purified EGFP in cell-free TX-TL with and without formate (20 mM) and Fdh (30.6 µM). EGFP was produced and purified from an EGFP encoded pET29b(+) vector in *E.coli* BL21(DE3) cells as described previously in Giaveri et al., (2024). To prepare calibrants, EGFP was first diluted in buffer containing 50 mM HEPES pH 7.8, 200 mM NaCl, and 10% (v/v) glycerol before adding 3.5 µL of each calibrant to a TX-TL reaction of total volume 35 µL. The 35 µL solution contained 21 µL PURE*frex* 1.0 mix (17.5 µL solution I, 1.75 µL solution II, and 1.75 µL solution III), 0.7 µL RNAse inhibitor and 8.75 µL liposome mix with or without formate and Fdh. The final assay contained 1 mM ADP, 10 mM KPO_4, 3 mg mL^{-1} Etf:qo-Cyt*bo*$_3$-F$_1$F$_O$-PLs, 1 µM NDH-2, and 5 mM NADH. After adding nuclease-free water to bring the final volume to 35 µL, calibrants were incubated at 32 °C in Infinite M plex microplate reader for 10 min. The plate reader parameters were the following: mode = fluorescence bottom reading, interval time = 1 min, λex = 488 nm, λem = 515 nm, number of flashes = 25, integration time = 20 ms, and gain = 100. Panel B represents the statistical mean of data collected between 5 and 10 min at each EGFP concentration.

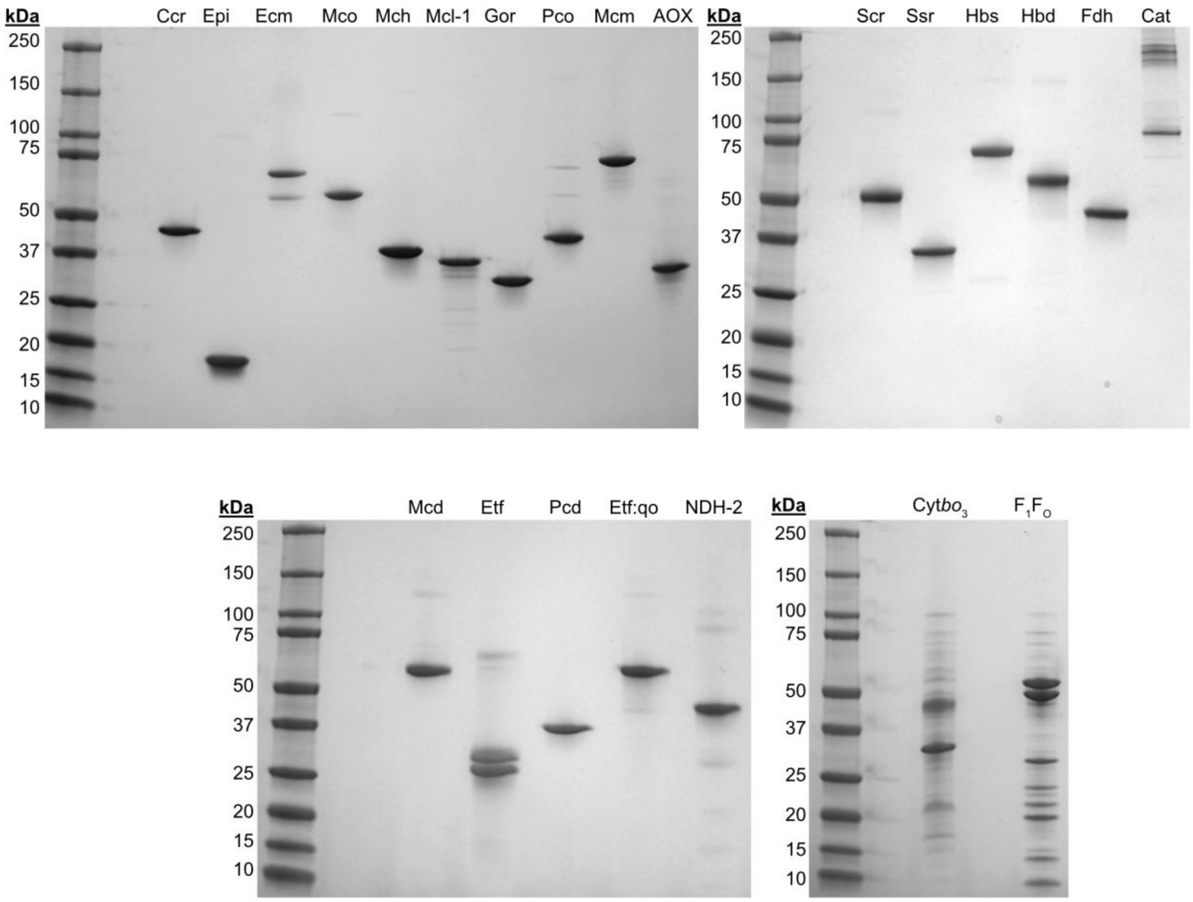

Figure S13. SDS-PAGE gels of all purified proteins used in this study. Expected molecular mass of each protein/subunit are as follows: Ccr (49.0 kDa), Epi (16.8 kDa), Ecm (73.7 kDa), Mco (62.2 kDa), Mch (39.7 kDa), Mcl-1 (36.8 kDa), Gor (35.7 kDa), Pco (50.1 kDa), Mcm (77.0 kDa), AOX (37.6 kDa) Scr (51.5 kDa), Ssr (39.2 kDa), Hbs (78.1 kDa), Hbd (60.5 kDa), Fdh (46.4 kDa), Cat (84.1 kDa), Mcd (62.3 kDa), Etf (EtfA: 30.9 kDa EtfB: 26.8 kDa), Pcd (42.9 kDa), Etf:qo (59.6 kDa), NDH-2 (48.6 kDa), Cyt*bo*$_3$ (I: 74.4 kDa, II: 34.9 kDa, III: 22.6 kDa, IV:12 kDa), F_1F_O (a: 30.3 kDa, b: 17.2 kDa, c: 8.8 kDa, α: 55.3 kDa, β: 50.3 kDa, γ: 31.6 kDa δ: 19.3 kDa, ε: 15.0 kDa). For multi-subunit complexes, individual bands were not assigned to specific subunits as no further identification was performed.

Supplementary note

Calculation of coupling efficiency of proteoliposomes

The theoretical efficiency of the artificial respiratory chain can be calculated as described. The F_1F_O-ATP synthase from *E. coli* has a *c*-ring of 10 subunits and will require 10 H^+ for one full rotation to synthesise 3 ATP molecules. In our system the bo_3 oxidase is the only proton pump and will pump $2H^+/2e^-$ to the liposome lumen as well as releasing an additional $2H^+/2e^-$ during ubiquinol oxidation. When conserving the electrons through one oxidation step of the CETCH cycle, a stoichiometry of $4H^+/2e^-$ is expected in fully coupled conditions. This would generate 1.2 ATP per methylsuccinyl-CoA oxidation. In our system where 5 mM crotonyl-CoA is transformed for the production of ~54 µM ATP through the Mcd-catalysed step, the efficiency of our system can be calculated at 0.9% of the possible maximum ATP yield.

Table S1. List of enzymes used in this study

Name	Full name	Origin	Source	Reference
Ccr	crotonyl-CoA carboxylase/reductase	*M. extorquens*	pTE71	Schwander et al. (2016)
Epi	methylmalonyl-/ethylmalonyl-CoA epimerase	*C. sphaeroides*	pTE45	Schwander et al. (2016)
Ecm	ethylmalonyl-CoA mutase	*C. sphaeroides*	pTE33A	Schwander et al. (2016)
Mco	methylsuccinyl-CoA oxidase	*C. sphaeroides*	pTE813	Schwander et al. (2016)
Mcd	methylsuccinyl-CoA dehydrogenase	*C. sphaeroides*	pTE801	Miller et al. (2020)
Etf	electron transfer flavoprotein	*C. sphaeroides*	pTE392	Miller et al. (2020)
Etf:qo	electron transfer flavoprotein:quinone oxidase	*C. sphaeroides*	pTE7000	This work
Mch	mesacony-CoA hydratase	*C. sphaeroides*	pMCH_Rs	Schwander et al. (2016)
Mcl-1	β-methylmalyl-CoA lyase	*C. sphaeroides*	pMCL1 Rs JZ 03	Schwander et al. (2016)
Gor	glycolate reductase	*G. oxydans*	pTE2124 Gox1125	Pandi et al. (2022)
Pco	propionyl-CoA oxidase	*A. thaliana*	pTE825	Schwander et al. (2016)
Mcm	methylmalonyl-CoA mutase	*C. sphaeroides*	pTE46	Schwander et al. (2016)
Scr	succinyl-CoA reductase	*C. kluyveri*	pTE380	Schwander et al. (2016)
Ssr	succinic semialdehyde reductase	*H. sapiens*	P2BP1	Schwander et al. (2016)
Hbs	4-hydroxybutyryl-CoA synthetase	*N. maritimus*	pNMAR_0206	Schwander et al. (2016)
Hbd	4-hydroxybutyryl-CoA dehydratase	*N. maritimus*	pTE393	Schwander et al. (2016)
Cat	catalase	*E. coli*	ASKA JW1721	Schwander et al. (2016)
Fdh	formate dehydrogenase	*M. vaccae*	mutMycFDH	Schwander et al. (2016)
AOX	alternative ubiquionl oxidase	*T. brucei brucei*	pET15b-aox	Jones et al. (2016)
bo$_3$ oxidase	cytochrome *bo*$_3$ oxidase	*E. coli*	pETcyo-βHis	von Ballmoos et al (2016)
F_1F_O-ATPase	F_1F_O-ATP synthase	*E. coli*	pBWU13-βHis	von Ballmoos et al (2016)
NDH-2	Alternative NADH dehydrogenase	*E. coli*	pTE7001	This work
Pcd	propionyl-CoA dehydrogenase	*C. sphaeroides*	pTE605	Peter et al. (2016)
Ca	carbonic anhydrase	bovine erythrocytes	Sigma Aldrich (C3934)	Commercial
CK	creatine kinase	rabbit muscle	Roche (10127566001)	Commercial

Table S2. Assay conditions for figures 1-4. The components that are highlighted were varied/exchanged during the experiment.

Component (units in parentheses)	Fig. 1D	Fig. 1E	Fig. 2B,C	Fig. 2D	Fig. 3B,C	Fig. 3D	Fig. 4B	Fig. 4C	Fig. 4D
HEPES, pH 7.8 (mM)	50	50	75	75	75	75	50	75	75
KCl (mM)	50	50	–	–	–	–	50	–	–
$MgCl_2$ (mM)	10	10	12.5	12.5	12.5	12.5	10	12.5	12.5
$KHCO_3$ (mM)	–	–	2.5	2.5	2.5	2.5	2.5	–	2.5
Formate (mM)	–	–	20	20	20	20	20	3.75	40
CoA (mM)	–	–	0.4	0.4	–	–	–	–	0.4
ADP (mM)	–	–	–	–	2	0	2	2	0.2
KPO_4 (mM)	–	–	–	–	10	10	10	10	10
ATP (mM)	–	–	3	3	–	1	–	–	–
NADPH (mM)	–	–	3.75	3.75	3.75	3.75	–	–	3.75
NADH (mM)	–	–	–	–	–	–	5	5	5
CP (mM)	–	–	60	60	–	–	–	–	–
PLs (mg mL^{-1})	1	1	1	1	3	1	3	3	1
GramA (mg mL^{-1})	–	–	–	–	0.1	0.1	0.1	0.1	0.1
Ap5A (mM)	–	–	–	–	0.5	0.5	0.5	0.5	0.5
Ccr (µM)	–	–	1.9	1.9	1.9	1.9	–	–	1.9
Epi (µM)	0.7	–	0.7	0.7	0.7	0.7	–	0.7	0.7
Ecm (µM)	1.4	–	1.4	1.4	1.4	1.4	–	1.4	1.4
Mco (µM)	10	–	26	0–55	–	26	–	–	26
Mcd (µM)	2	–	4	0–15	3	10	–	3	10
Etf (µM)	2	2	4	0–15	3	10	–	3	10
Etf:qo (µM)	1	1	4	0–15	–	–	–	–	–
Mch (µM)	–	–	0.3	0.3	0.3	0.3	–	–	0.3
Mcl-1 (µM)	–	–	3.6	3.6	3.6	3.6	–	–	3.6
Gor (µM)	–	–	5	5	5	5	–	–	5
Pco (µM)	–	1	3.1	3.1	3.1	3.1	–	–	3.1
Pcd (µM)	–	20	25	–	–	–	–	–	–
Mcm (µM)	–	–	2.9	2.9	–	2.9	–	–	2.9
Scr (µM)	–	–	3.5	3.5	–	3.5	–	–	3.5
Ssr (µM)	–	–	1.7	1.7	–	1.7	–	–	1.7
Hbs (µM)	–	–	0.5	0.5	–	0.5	–	–	0.5
Hbd (µM)	–	–	0.7	0.7	–	0.7	–	–	0.7
Cat (µM)	–	–	3.3	3.3	3.3	3.3	–	3.3	3.3
Fdh (µM)	–	–	30.6	30.6	30.6	30.6	30.6	30.6	30.6
Ca (µM)	–	–	0.07	0.07	0.07	0.07	–	–	0.07
CK (µM)	–	–	0.8	0.8	–	–	–	–	–
AOX (µM)	0.26	0.26	0.26	0.26	–	–	–	–	–
NDH-2 (µM)	–	–	–	–	–	–	1	1	1
Propionyl-CoA (mM)	–	0.1	0.1	0.1	–	1	–	–	0.1
Ethylmalonyl-CoA (mM)	0.1	–	–	–	–	–	–	5	–
Crotonyl-CoA (mM)	–	–	–	–	5	–	–	–	–

Table S3. Protein sequences for Etf:qo and NDH-2 used in this study.

Protein	Sequence
Etf:qo	MGSSHHHHHHSSGLVPRGSHTEQTPREQMEYDVVIVGAGPSGLSAAIRLKQLDPDLSVVLLEKGSEVGAHI<br>LSGAVLDPAGLNALIPDWKEKGAPLNTPVTEDHFFVLTPAGQTMLPSWPVPKLMDNHGNYVVSMGNVCRW<br>LATQAEELGVEIFPGMSCSELVYGENGEVRGVVAGEFGKNADGTPGPSYEPGMELLGKYVMLAEGVRGSL<br>SKQVMEKFDLRKGYGPQKYGLGMKEIWEIDPAKHKPGRIWHTMGWPLGKNAGGGSFIYHAENNQVFIGLV<br>VHLNYENPHLYPYQEFQRFKHHPMVAELLKGGKRVAYGARAISEGGYQSLPKMVFPGGALLGCSAGMVNV<br>PRIKGNHNAMLSGKAAAEAAHAAIKAGRQSDELSDYEQSVRKGPIGQDLWKVRNVKPIWSHLGLYASMALG<br>GLDMWTNSLFNFSFFGTMKHHQTDAADTKPARDFAPIDYPKPDGVLSFDRLTNVAFSFTNHEESQPAHLKL<br>KDPAIPIAVNLPKYAEPAQRYCPAGVYEVVSEAGRDPRFVINFQNCVHCKTCDIKDPSQNIDWTTPQGGDG<br>PNYPNM*
NDH-2	MGMTTPLKKIVIVGGGAGGLEMATQLGHKLGRKKKAKITLVDRNHSHLWKPLLHEVATGSLDEGVDALSYLA<br>HARNHGFQFQLGSVIDIDREAKTITIAELRDEKGELLVPERKIAYDTLVMALGSTSNDFNTPGVKENCIFLDNP<br>HQARRFHQEMLNLFLKYSANLGANGKVNIAIVGGGATGVELSAELHNAVKQLHSYGYKGLTNEALNVTLVE<br>AGERILPALPPRISAAAHNELTKLGVRVLTQTMVTSADEGGLHTKDGEYIEADLMVWAAGIKAPDFLKDIGGL<br>ETNRINQLVVEPTLQTTRDPDIYAIGDCASCPRPEGGFVPPRAQAAHQMATCAMNNILAQMNGKPLKNYQY<br>KDHGSLVSLSNFSTVGSLMGNLTRGSMMIEGRIARFVYISLYRMHQIALHGYFKTGLMMLVGSINRVIRPRLK<br>LHLEHHHHHH*

Table S4. Instrument parameter settings for glycolate LC-MS/MS quantification analysis

Name	Precursor Ion	Product Ion	Collision energy [V]	Fragmentor Voltage [V]	Cell Accelerator Voltage [V]	Dwell time [msec]	Polarity
Glycolate	75.2	75.2<br>47.2	0<br>9	80	5	150	Negative
$^{13}C_2$-Glycolate (ISTD)	77.2	77.2<br>48.2	0<br>9	80	5	150	Negative

Table S5. Instrument parameter settings for ATP and glycolate LC-MS/MS quantification analysis

Name	Precursor Ion	Product Ion	Collision energy [V]	Fragmentor Voltage [V]	Cell Accelerator Voltage [V]	Dwell time [msec]	Polarity
ATP	505.9	407.9<br>158.8	21<br>28	380	5	130	Negative
Glycolate	75	75<br>47	0<br>9	380	5	130	Negative
$^{13}C_2$-Glycolate (ISTD)	77	77<br>48	0<br>9	380	5	130	Negative

EGFP gBlock and forward and reverse primers

GCACCATCAGCCAGAAAACCGAACCAGCCAGAAAACGACCTTTCTGTGGATCTTAAGGCTAGAGTACTAATACGACTCACT
ATAGGGAGACCACAACGGTTTCCCTCTAGAAATAATTTTGTTTAACTTAAGAAGGAGGAAAAAAAAATGGTCTCTAAAGGTG
AAGAATTATTCACTGGTGTTGTCCCAATTTTGGTTGAATTAGATGGTGATGTTAATGGTCACAAATTTTCTGTCTCCGGTGAA
GGTGAAGGTGATGCTACTTACGGTAAATTGACCTTAAAATTTATTTGTACTACTGGTAAATTGCCAGTTCCATGGCCAACCT
TAGTCACTACTTTAACTTATGGTGTTCAATGTTTTTCTAGATACCCAGATCATATGAAACAACATGACTTTTTCAAGTCTGCC
ATGCCAGAAGGTTATGTTCAAGAAAGAACTATTTTTTTCAAAGATGACGGTAACTACAAGACCAGAGCTGAAGTCAAGTTTG
AAGGTGATACCTTAGTTAATAGAATCGAATTAAAAGGTATTGATTTTAAAGAAGATGGTAACATTTTAGGTCACAAATTGGAA
TACAACTATAACTCTCACAATGTTTACATCATGGCTGACAAACAAAAGAATGGTATCAAAGTTAACTTCAAAATTAGACACAA
CATTGAAGATGGTTCTGTTCAATTAGCTGACCATTATCAACAAAATACTCCAATTGGTGATGGTCCAGTCTTGTTACCAGAC
AACCATTACTTATCCACTCAATCTGCCTTATCCAAAGATCCAAACGAAAAGAGAGACCACATGGTCTTGTTAGAATTTGTTA
CTGCTGCTGGTATTACCTTAGGTATGGATGAATTGTACAAACACCACCATCATCACCACTAATAACGACTCAGGCTGCTACC
TAGCATAACCCCTTGGGGCCTCTAAACGGGTCTTGAGGGGTTTTTTGGCAGGAAAGAACATGTGAGCAAAAGG

Forward Primer 5’-GATCTTAAGGCTAGAGTAC-3’

Reverse Primer 5’-CAAAAAACCCCTCAAGAC-3’
